# Genome-wide analysis of the soybean CRK-family and transcriptional regulation by biotic stress signals triggering plant immunity

**DOI:** 10.1371/journal.pone.0207438

**Published:** 2018-11-15

**Authors:** Leonardo Delgado-Cerrone, Alfonso Alvarez, Eilyn Mena, Inés Ponce de León, Marcos Montesano

**Affiliations:** 1 Departamento de Biología Molecular, Instituto de Investigaciones Biológicas Clemente Estable, Montevideo, Uruguay; 2 Laboratorio de Fisiología Vegetal, Centro de Investigaciones Nucleares, Facultad de Ciencias, Universidad de la República, Montevideo, Uruguay; ICAR-National Research Centre on Plant Biotechnology, INDIA

## Abstract

Cysteine-rich receptor-like kinases (CRKs) are transmembrane proteins that exhibit ectodomains containing the domain of unknown function 26 (DUF26). The CRKs form a large subfamily of receptor-like kinases in plants, and their possible functions remain to be elucidated. Several lines of evidence suggest that CRKs play important roles in plant defense responses to environmental stress, including plant immunity. We performed a genome-wide analysis of CRK encoding genes in soybean (*Glycine max*). We found 91 *GmCRKs* distributed in 16 chromosomes, and identified several tandem and segmental duplications, which influenced the expansion of this gene family. According to our phylogenetic analysis, GmCRKs are grouped in four clades. Furthermore, 12% of the members exhibited GmCRKs with a duplicated bi-modular organization of the ectodomains, containing four DUF26 domains. Expression analysis of *GmCRKs* was performed by exploring publicly available databases, and by RT-qPCR analysis of selected genes in soybean leaves responding to biotic stress signals. *GmCRKs* exhibited diverse expression patterns in leaves, stems, roots, and other tissues. Some of them were highly expressed in only one type of tissue, suggesting predominant roles in specific tissues. Furthermore, several *GmCRKs* were induced with PAMPs, DAMPs and the pathogens *Phakopsora pachyrhizi* and *Phytophthora sojae*. Expression profiles of several *GmCRKs* encoding highly similar proteins exhibited antagonist modes of regulation. The results suggest a fine-tuning control of *GmCRKs* transcriptional regulation in response to external stimuli, including PAMPs and DAMPs. This study offers a comprehensive view of the GmCRKs family in soybean, and provides a foundation for evolutionary and functional analysis of this family of plant proteins involved in the perception of pathogens and activation of plant immunity.

## Introduction

Plants activate the immune system by perception of pathogen associated molecular patterns (PAMPs)/microbe-associated molecular patterns (MAMPs), such as bacterial flagellin or fungal chitin, or by perception of plant damage associated molecular patterns (DAMPs), such as oligogalacturonides (OGs). Surface-localized pattern recognition receptors (PRRs) mediate the recognition of elicitors, and activate intracellular signaling leading to pattern triggered immunity (PTI) [1]. PTI responses include an extracellular burst of reactive oxygen species (ROS), Ca^2+^ influx, activation of kinase-signaling cascades, and transcriptional reprogramming [2]. Host-adapted pathogens must overcome PTI to cause disease, and they suppress PTI by secretion of proteins called effectors into the apoplast or inside the plant host cells [3]. Plant resistance proteins recognize pathogen effectors and activate effector-triggered immunity (ETI) that usually is a stronger plant response leading to programmed cell death at the site of infection [4].

PRRs are either receptor-like kinases (RLKs) or receptor-like proteins (RLPs). RLKs contain an ectodomain involved in ligand binding, and a single-pass transmembrane domain connecting to an intracellular kinase domain. RLPs have similar structural organization than RLKs but lack the kinase domain [1]. The ectodomain of RLKs contains highly diverse protein domains used for subclassification of the RLK family [5–7]. For example, the leucine rich repeat (LRR)-RLK FLS-2 from *Arabidopsis* binds bacterial flagellin (flg22) and triggers plant immunity leading to resistance to bacterial phytopathogens [8, 9]. Upon flagellin perception, FLS2 integrates hetero-oligomeric receptor signaling complexes with other RLKs such as BAK1 [10]. In contrast, the *Arabidopsis* LysM-RLK CERK1 ectodomain binds fungal chitin, leading to CERK1 homo-dimerization that integrates a plasma membrane-signaling complex triggering plant immunity [11]. However, in rice chitin perception involves a hetero-oligomeric receptor complex containing both LysM-RLK OsCERK1 and RLP-CEBiP [1]. Furthermore, *Arabidopsis* Wak1-RLK perceives damage by detecting specifically molecules such as OGs [12].

The cysteine-rich receptor-like kinases (CRKs), defined by the presence of an ectodomain with cysteine-rich modules containing DUF26 (Domain of Unknown Function 26; PF01657; stress-antifungal domain), constitute a large subfamily of the plant RLK family [6,13,14]. The DUF26 domain contains a conserved configuration of three cysteines (C-X8-C-X2-C), suggested as a potential target of redox regulation that proposed the CRKs as possible candidates for ROS perception [15]. There are more than 40 CRKs in the *Arabidopsis* genome, and most of them exhibit ectodomains with a bi-modular organization, each module containing one DUF26 domain [5, 6, 16]. Several lines of evidence suggest a role for AtCRKs in *Arabidopsis* immunity. *AtCRKs* expression patterns have been analyzed in response to ROS, PAMPs and other treatments, and a model for CRKs transcriptional regulation by plant hormones and ROS perception has been proposed [16]. Large-scale phenomics analysis of a collection of *Arabidopsis crk* T-DNA insertion lines identified primary and fine-tuning roles for AtCRKs related to oxidative stress [17]. Moreover, *CRK* overexpression could lead to biotic and abiotic stress resistance. For example, overexpression of *AtCRK13* in *Arabidopsis*, resulted in enhanced resistance to *Pseudomonas syringae* [18], while the *Arabidopsis crk5* mutant analysis reveals a role in plant growth and defense to ultraviolet radiation, the overexpression of *AtCRK5* enhanced abscisic acid (ABA) sensitivity and drought tolerance [19,20]. CRKs also integrate immune receptor complexes. In recent studies, *AtCRK28* overexpression increased *Arabidopsis* disease resistance to *Pseudomonas syringae*, while the transient overexpression of *AtCRK28* in *Nicotiana benthamiana* induced cell death, which required intact cysteine residues and a conserved kinase active site [21]. Furthermore, AtCRK28 induced cell death required BAK1, and was detected in FLS2-BAK1 immune receptor complex [21].

The soybean genome underwent two rounds of whole genome duplication, approximately 59 and 13 million years ago, and nearly 75% of the genes are present in multiple copies [22]. The soybean RLK family encodes more than 1400 RLKs [7]; while more than 450 belong to the LRR-RLK sub family [23]. In the present study, a genome-wide analysis for CRK-RLKs was performed in soybean (*Glycine max*), and 91 GmCRKs were identified. Phylogenetic analysis of the GmCRKs was performed, and the organization of the ectodomains, and gene localization were defined. Soybean genome analysis indicated that tandem duplications and segmental duplications influenced the expansion of *GmCRKs* evolution in *G*. *max*. Transcriptional regulation of *GmCRKs* was analyzed by exploring public available databases and the expression analysis of selected genes by RT-qPCR was performed. Our results reveal novel types of modular DUF26 organization of the ectodomains for the CRK family. This work provides a framework for further functional characterization of GmCRKs.

## Materials and methods

### Database search for soybean CRK proteins

A workflow for semimanual extraction, annotation, and verification of plant receptor kinases from annotated gene models was used [24]. First, a search for soybean CRK proteins was performed using BLASTP in NCBI (http://www.ncbi.nlm.nih.gov/) and the Phytozome *Glycine max* version 12.1 (http://www.phytozome.net /cgi-bin/ gbrowse/ soybean/) databases, using as query CRK sequences from *Solanum tuberosum* previously identified [25]. Putative CRK proteins were analyzed for the existence of the relevant domains (extracellular cysteine rich domain and kinase domain), and all Hidden Markov Models (HMM) protein profiles contained in the Pfam-A model were downloaded from Pfam database [26], and explored for the identification of CRKs using HMMER3 Package [27]. The protein domains found were illustrated with the DoMosaics program [28]. Hydrophobicity plots were used to analyze the secondary structure of the GmCRK members as well as the presence of transmembrane regions [29]. In order to check the domains integrity, multiple sequence alignments (MSA) were performed with MUSCLE [30], available within the MEGA6 software [31]. All CRK proteins were manually inspected to ensure the presence of these three important domains (ectodomain containing PF01657, single-pass transmembrane domain, and kinase domains PF00069 or PF07714), and putative CRK proteins lacking some of these domains were discarded. Most of the putative CRK proteins were retrieved from the Phytozome database, and in those cases where incomplete sequences or domains were found, the corresponding sequences from NCBI database were used. Identification numbers of all GmCRK are listed in S1 Table. In order to identify CRKs from other plants species, including *Medicago truncatula* (*M*. *truncatula*) and *Phaseoulus vulgaris* (*P*. *vulgaris*), a similar approach as mentioned for soybean was used. The corresponding CRKs for other plants are listed in S2 and S3 Tables. Exon-intron structures of the *CRK* genes were based on the genome and coding sequences, and were identified using the Gene Structure Display Server (GSDS: http://gsds.cbi.pku.edu.cn/) online tool [32]. Segmental block duplications between chromosomes were detected in the PLAZA 4.0 online platform (https://bioinformatics.psb.ugent.be/plaza/versions/plaza_v4_dicots/) [33]. The block duplications were established by analyzing the multiplicons table for *Glycine max*. According to the anchor point positions for each gene in the table, block duplications in chromosomes were established and drawn. Putative candidates for alternative splicing events for each *CRK* gene were obtained from Phytozome and NCBI.

### Phylogenetic analysis of soybean CRKs

Full-length amino acid sequences from putative soybean CRKs were retrieved from databases. Sequences were aligned with the MUSCLE program [30], available within the MEGA6 software [31]. The amino acidic substitution model was determined with MEGA6, and the JTT model [34], assuming discrete gamma (+G) with a fraction of evolutionarily invariable sites (+I), was chosen for subsequent phylogenetic analysis. Construction of the phylogenetic tree was done by Maximum Likehood analysis using the RAxML software [35], performing 1000 rapid bootstrap replicates [36] with the MRE-based Bootstopping criterion [37].

### Cis-regulatory elements identification and *in silico* expression profiles

The 1,000 bp upstream of the transcription start site of all soybean CRK genes were obtained from Phytozome v12.1 (http://www.phytozome.net/), and the potential cis-regulatory elements were identified using PlantCARE, http://bioinformatics.psb.ugent.be/webtools/plantcare/html [38]. To evaluate the expression of soybean CRK-encoding genes in different tissues, the fragments per kilobase of transcript per million mapped reads (FPKM) values for each CRK gene were retrieved from Phytozome v12.1 database. RNA-seq based expression data from PAMPs- (flg22+chitin) treated soybean tissues and microarray based expression data for pathogen-infected tissues were obtained from the Genevestigator (http://www.genevestigator.ethz.ch) database [39]. Signal intensities from treatment groups (flg22+chitin or pathogens) were compared to signal intensities from the corresponding control samples generating log_2_-ratios. For flg22+chitin treatment (30 min) data from two parental soybean genotypes, one with a strong PTI response (LD00-2817P; [40]) and one with a weak PTI response (LDX01-1-65; [41]) were used [42]. For *Phakopsora pachyrhizi* (*P*. *pachyrhizi; Hawaii 94–1* isolate), we used data from genotypes Williams (cultivar PI548631; susceptible to isolate *Hawaii 94–1*) and PI459025B (resistant to isolate *Hawaii 94–1*). For *Phytophthora sojae* (*P*. *sojae*; *1*.*S*.*1*.*1* isolate), data from genotypes Sloan (susceptible) and Conrad (resistant) were used [43]. The heat maps were generated using the Heatmapper server (www.heatmapper.ca/expression). To analyze the expression profiles of CRKs from *M*. *truncatula* and *P*. *vulgaris* in different tissues and conditions, FPKM values were retrieved from Phytozome v12.1 database.

### Gene Ontology analysis

A list of 89 *GmCRK* genes (UniProt IDs) was subjected to Gene Ontology (GO) functional analysis using Singular Enrichment Analysis (SEA) method by agriGOv2.0 tool (http://systemsbiology.cau.edu.cn/agriGOv2/) [44]. GmCRK32 and GmCRK54 were not included in the analysis since UniProt ID could not be identified. The results were obtained with the background entry identifier (UniProt 2016), and visualized by directed acyclic graph.

### Plant material and treatments

Soybean (*Glycine max*) cultivar Williams (PI 548631, USDA Agricultural Research Service, Soybean germplasm collection, seed source 13U-9280) was used for all assays. Three seeds were planted in a 10 cm diameter pot filled with a mix of soil and vermiculite at a rate of 3:1. Soybean seedlings were grown in a growth room under a 16 h light/8 h dark photoperiod at 24°C. For all experiments, the second trifoliolate leaves of 4-week-old plants at V3 were detached and vacuum infiltrated with MilliQ water for 3 min as previously described [42]. Discs from infiltrated leaves were cut using a cork borer and incubated in petri dishes with MilliQ water in the dark overnight (ON). Water was then removed from each petri dish and replaced for a 5 mL solution containing 1 μM flg22, 100 μg/mL chitin (Yaizu Suisankagaku Industry), 1 mM di-galacturonic acid (OG) (Sigma-Aldrich), or 1 mM Xanthine-0.1 U ml^-1^ Xanthine Oxidase (X/XO) (Sigma-Aldrich). Water was used as control. After 30 min and 90 min leaf discs were frozen in liquid nitrogen and stored at -80°C.

### ROS production in leaf tissues

ROS production was measured in 0.4 cm^2^ leaf discs using hydrogen peroxide-dependent chemical luminescence of luminol [45]. Each disc from infiltrated leaves was incubated in 200 μL autoclaved MilliQ water in a 96-well plate in the dark ON. Water was removed and replaced with 100 μL solution containing 2 mM MES pH = 7, 200 μM luminol (Sigma-Aldrich), 0.02 mg/mL horseradish peroxidase (Sigma-Aldrich), and 1 μM flg22, 100 μg/mL chitin, 1 mM OG, or 1 mM Xanthine-0.1 U ml^-1^ Xanthine oxidase. Water was used as control. After treatments, luminescence detection assay was performed for at least 45 min, using a Varioskan Flash (Thermo Fisher Scientific). Each treatment was performed with six biological and three technical replicates. Values obtained from water treatment were used to normalize the data.

### RNA extraction, cDNA synthesis and real-time quantitative PCR

Time points for treatments were selected based on potato *CRKs* expression to bacterial elicitors and OGs [25]. Concentration of OGs, flg22 and chitin, was used as previously described [25,42]. Total RNA was extracted from 15 leaf discs of 1 cm^2^ treated with flg22, chitin, OG, X/XO or water for 30 and 90 min, using RNeasy Plant Mini Kits according to manufacturer’s instructions (Qiagen, Germany). For cDNA synthesis, 4 μg of total RNA were treated with DNase I (Thermo Scientific) and cDNA was synthesized from total RNA using RevertAid Reverse transcriptase (Thermo Scientific) and oligo (dT) according to the manufacturer’s protocol. RT qPCR was performed in a 96-well thermocycler (New Applied Biosystems QuantStudio 3) using the QuantiNova Probe SYBR Green PCR Kit (Qiagen, Germany). Each 20 μL reactions contained 10 μL of SYBR Green PCR Master mix (2X), 0.7 μM primers mix and 1 μL of template cDNA. The thermocycler was programmed to run for 2 min at 95 ^o^C, followed by 40 cycles of 15 s at 94 ^o^C and 20 s at 60 ^o^C. The gene *Ef1* was used as the internal control, and expression in water-treated tissues was used as the calibrator, with the expression level set to one. Each pair of primers were specific to each GmCRK, and the amplification efficiencies were all greater than 90%. Relative expression was determined using the 2^-ΔΔCt^ method [46]. Each data point is the mean value of three biological replicates, each consisting of 15 leaf discs taken from the second trifoliolate leaves from 3 different plants. Two technical replicates were used for each sample. An unpaired t-test was performed to determine the significance for quantitative gene expression analysis using GraphPad Prism software v5.0. P values <0.05 were considered statistically significant. The following symbols were used: *P < 0.05; **P < 0.01; ***P < 0.001. The primers used for qPCR analyses are provided in S4 Table.

## Results

### Genome-wide identification, phylogenetic analysis, and gene structure of the CRK family in soybean

**Fig 1:**
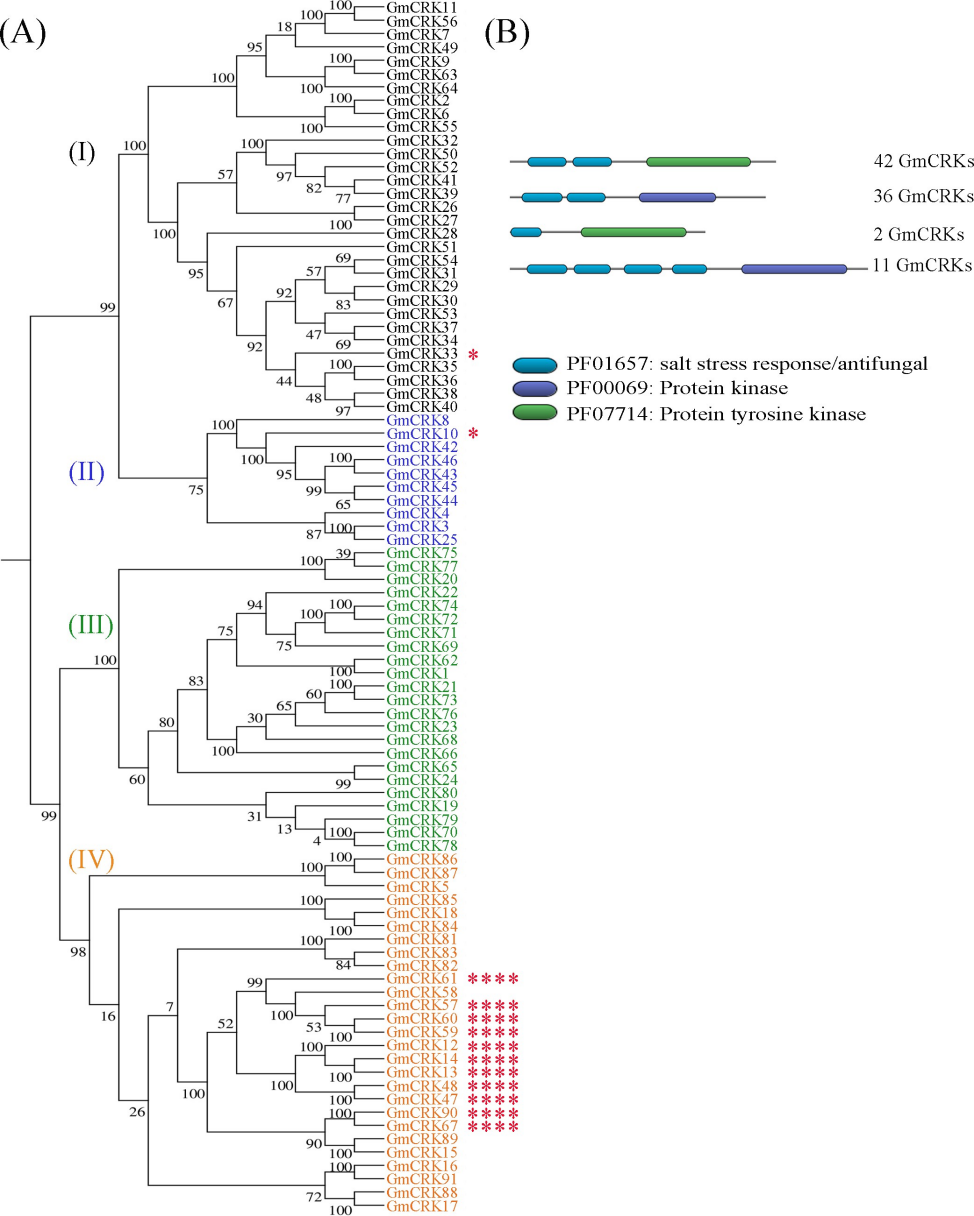
Phylogenetic tree and domains in GmCRK. **(A)** Phylogenetic tree constructed based on the amino acid sequences of the 91 putative GmCRK proteins. Clades I, II, III, and IV and the corresponding GmCRK proteins are indicated with different colors. **(B)** Pfam domains present in soybean GmCRK predicted proteins. GmCRKs with one DUF26 domain are marked by one red asterisk, and GmCRKs with four DUF26 domains with four red asterisks.

To identify putative CRK proteins in the soybean genome of *G*, *max*, BLASTP searches were performed at the NCBI and Phytozome *Glycine max* version 12.1 servers. In order to verify that the coding amino acid sequences have the typical cysteine rich ectodomain and intracellular kinase domains, all candidates were analyzed by scanning with HMMER3 against all the HMM protein profiles contained in the Pfam-A model. The presence of a single-pass transmembrane region was verified in all putative CRKs by hydrophobicity plots and multiple sequence alignments (MSA). A total of 91 soybean CRKs were identified, which were designated as GmCRK1 through GmCRK91 according to their top-to-bottom positions on chromosomes from 1 to 20 (S1 Table). One additional gene, *Glyma20G138700*, was excluded from the analysis since it was classified as a pseudogene at NCBI, and no transmembrane domain could be identified by hydrophobicity plot analysis. The 91 *GmCRKs* were mapped to the 20 chromosomes, and their genomic distribution and chromosomal localization defined (S1 Table). The results show that *GmCRKs* are not equally distributed across the 20 chromosomes. Chromosome 20 contains most members (27 *GmCRKs*; 29.67%), followed by chromosome 11 (17 *GmCRKs*; 18.68%), chromosome 18 (13 *GmCRKs*; 14.29%), and chromosome 10 (10 *GmCRKs*; 10.99%). Some chromosomes did not contain any *GmCRK*, including chromosomes 3, 7, 12 and 14. The rest of the chromosomes exhibited between 1 to 5 *GmCRKs* per chromosome. The encoded proteins varied from 476 to 948 amino acid residues, and exhibited an ectodomain with different modular organization of DUF26 domains (PFAM PF01657), and the kinase domain represented by PF00069 (Protein kinase) or PF07714 (Protein tyrosine kinase) (Fig 1; S1 Table). All GmCRKs ectodomains exhibited DUF26 domains that contain the classical C-X8-C-X2-C motif, except for GmCRK68, which exhibited a mutation in the last cysteine of the first translated DUF26 domain. Most GmCRK exhibit an ectodomain with two modules, each containing one DUF26 domain (78 GmCRKs, including GmCRK68), 2 GmCRKs contain only one DUF26 domain, and 11 GmCRKs exhibited 4 modules of DUF26 domains. While protein sequences of GmCRKs with ectodomains containing 2 modules of DUF26 range from 529–745 amino acids, GmCRKs with 4 DUF26 range from 903–948 amino acids, indicating that the latter possess bigger extracellular ectodomains with additional structural combinations of DUF26 domains for perception of stimuli. Thus, 85% of the GmCRKs exhibited ectodomains with a bi-modular organization of DUF26, while 12% have a duplicated bi-modular organization of DUF26.

To gain further insights into the phylogenetic relationship among the different members of the soybean CRK gene family, an unrooted phylogenetic tree was generated (Fig 1). The result shows that GmCRKs are distributed in 4 well supported clades (bootstrap values ≥75%). Clade I consists of 31 GmCRKs, clade II has 10 GmCRKs, and clades III and IV have 23 and 27 GmCRKs, respectively. GmCRKs from chromosome 11 and 18 are overrepresented in Clade I, including 16 of the 17 GmCRKs present in chromosome 11, and 7 of the 13 GmCRKs present in chromosome 18. Similarly, 15 of 27 GmCRKs from chromosome 20, and 6 of 10 GmCRKs of chromosome 10 are present in Clade III. GmCRKs from chromosome 20 are the most abundant in Clade IV, consisting of 12 GmCRKs. All clades have most GmCRKs with two modules of DUF26 domains. Clade III has only GmCRKs with ectodomains exhibiting two DUF26 domains, while clade I and II, each contain one GmCRK with one DUF26 domain (GmCRK33 and GmCRK10, respectively), and clade IV contains 11 GmCRKs with 4 modules of DUF26 domains (Fig 1; S1 Table). All *GmCRK* genes have several exons ranging from 6 to 9 exons. The ectodomain is encoded in most of the cases by one or two exons, the transmembrane domain is encoded only by one exon, and the intracellular domain by four or five exons (S1 Fig). The ectodomain of all GmCRKs with 4 modules, each containing one DUF26 domain, are encoded by only one exon. Furthermore, 43 *GmCRK* genes contained two to 11 alternative splicing variants, producing a variety of transcripts from a single *GmCRK* gene (S1 Table), which could increase functional diversity among GmCRKs. Taken together, the results show that the GmCRK family contains an extensive number of receptor kinases with a variety of ectodomains that exhibit structural characteristics based on different arrangements of DUF26 modules.

### CRKs duplication events in soybean

**Fig 2:**
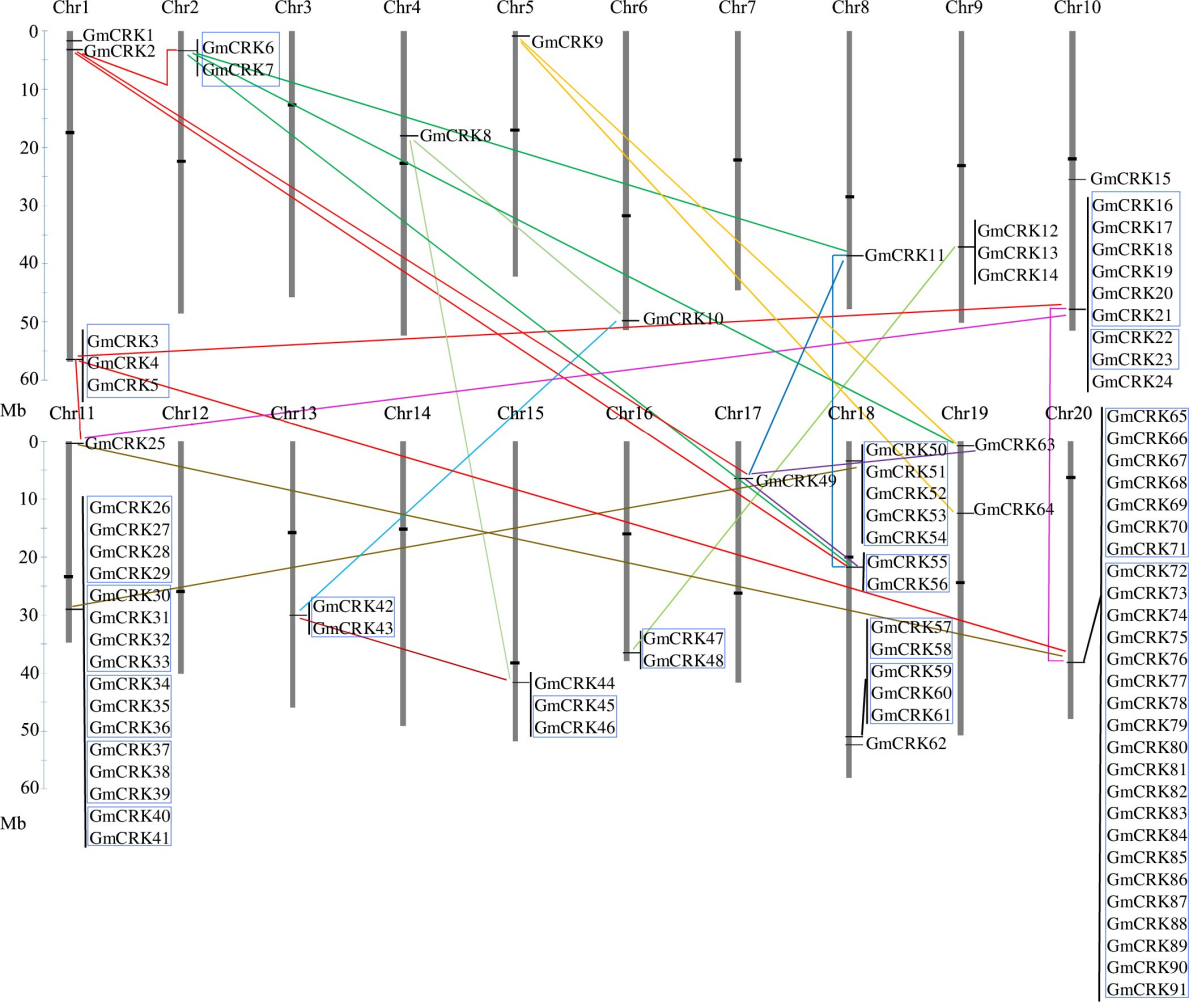
Chromosomal localization and gene duplication events of *GmCRKs*. The GmCRK genes were mapped to the 20 soybean chromosomes and those *GmCRKs* found on duplicated chromosomal segments are connected by lines. Blue outlined boxes represent tandemly duplicated genes. The chromosome numbers are indicated at the top of each bar and sizes of chromosomes are represented by the vertical scale. The scale is in megabases (Mb). The locations of centromeric repeats are shown as black lines over the chromosomes.

Gene duplication is an important event leading to gene family expansion and it contributes to the evolution of novel gene functions [47]. Several mechanisms, such as tandem duplications, segmental duplications, or even whole-genome duplications can lead to the expansion of gene families. To understand the potential expansion mechanism of the *CRK* gene family in soybean, segmental and tandem duplication events were analyzed. We detected 24 segmental duplications events distributed across all the chromosomes that contain *GmCRKs* (Fig 2). A high number of predicted *GmCRKs* have paralogous counterparts in regions of different chromosomes, including the large section between chromosome 11 and 18, and chromosome 10 and 20. When segmental duplication events were observed in more detail, we identified 9 pairs of *GmCRKs* paralogs of different chromosomes encoding highly similar proteins (≥91%), conforming a terminal node in the phylogenetic tree (bootstrap values ≥ 99%) (Fig 1; S5 Table). These pairs are distributed among the four clades. We also identified 74 *GmCRKs* located in tandem repeats in different chromosomes, with no or only one intervening annotated gene. These tandems were distributed in 18 subgroups, containing between two and 20 *GmCRK* members (S6 Table). The results show that in the 9 chromosomes with tandem duplications, 60 to 100% of the *GmCRKs* are distributed in tandems. Taken together, the results suggest that segmental and tandem duplications have contributed to the expansion of the *GmCRK* family in soybean.

### Cis-acting elements in *GmCRKs* and Gene Ontology enrichment analysis

To gain further insight into the possible roles played by GmCRKs in plant immunity, we analyzed the promoter regions of each member of this gene family. Based on the Phytozome version 12.1, putative cis-acting regulatory DNA elements in the promoter sequences (1,000 bp upstream of the translation start site) were analyzed with PlantCARE [38]. Potential biotic stress related elements were present in promoters of 76 *GmCRKs* (S7 and S8 Tables). A total of 50 *GmCRKs* promoter regions contain the TC-rich repeat element involved in defense and stress responsiveness, and 30 *GmCRKs* exhibited DNA elements related to wounding and pathogen responsiveness. Phytohormone responsive elements were present in the promoter region of 84 *GmCRKs*, including hormones involved in plant defense to biotic stress such as methyl jasmonate, salicylic acid, and ethylene. Furthermore, GmCRKs could also play important roles in abiotic stress responses, since 71 *GmCRKs* have cis-regulatory elements in their promoter region, mainly related to heat and drought stress. We also compared the promoter regions of each member of the segmental pairs identified (S5 Table). Only one segmental duplicated pair, *GmCRK3/GmCRK25*, exhibited the same promoter elements related to biotic stress, wounding and hormonal responsiveness, while in the other 8 pairs, some of the biotic stress and hormonal promoter elements were only present in one of the members of a pair (S8 Table). Thus, most *GmCRKs* members from segmental pairs encoding highly similar proteins, displayed differences in their promoter elements, suggesting that each member of a segmental pair could play specific roles in cells responding to environmental stimuli. These results suggest that GmCRKs could play important roles in plant stress perception and signaling, including abiotic stress and innate immunity. Consistently, Gene Ontology (GO) enrichment analysis (S2 Fig) confirmed that GmCRKs are mainly involved in protein phosphorylation and responses to multi-organism processes, biotic stimuli, and stress responses, including defense responses against other organisms.

### Expression patterns of *GmCRKs* in different plant organs and tissues

Based on publicly available information at the Phytozome database, we investigated the expression profiles of the 91 *GmCRKs* in different tissues and organs, including leaves, stems, roots, root hairs, shoot apical meristems, nodules, flowers, pods and seeds. The expression profiles were summarized against the phylogenetic tree (S3 Fig). The results show that *GmCRKs* exhibited distinct expression patterns among tissues/organs, and in each type of tissue different numbers of *GmCRKs* were expressed. We found a high number of *GmCRKs* expressed in leaves (50), nodules (43), roots (41), flowers (36), stems (34), and root hairs (32) (S3 Fig, S9 Table). While 21 *GmCRKs* showed low (FPKM ≤1) or undetectable expression (FPKM = 0) in all the tissues analyzed, 6 *GmCRKs* showed higher expression (˃1 FPKM) in all tissues, including *GmCRK5*, *6*, *17*, *18*, *84*, and *85*. Furthermore, 41 *GmCRKs* are expressed in more than three types of tissues. A few *GmCRKs* were highly expressed in one type of tissue (FPKM ≥3), while in the rest of the tissues their expression was low or undetectable. For example, *GmCRK14*, *44* and *60* were specific for leaves, while *GmCRK58* was specific for roots, suggesting that these genes could play specific functions in these particular tissues.

Conserved co-expression profiles between members of the 9 pairs representing segmentally duplicated *GmCRKs* encoding highly similar proteins (≥91–98% similarity; S5 Table) were analyzed. We found that only the pair *GmCRK16/GmCRK91* exhibited similar transcript levels for both members in 8 plant tissues (S4 Fig). In contrast, none of the other 8 *GmCRK* pairs with high protein similarity, exhibited similar expression patterns in the different tissues. In 4 pairs of *GmCRKs* from different clades, *GmCRK11*/*GmCRK56* from clade I, *GmCRK43*/*GmCRK46* from clade II, *GmCRK21*/*GmCRK73* from clade III, and *GmCRK18*/*GmCRK84*, from clade IV, one of the members of each pair always exhibited higher transcript levels than its counterpart, and the difference increased in specific plant tissues. Interestingly, members of the pair *GmCRK21*/*GmCRK73* exhibited different kinase domains, suggesting that despite the high protein sequence similarity, they could perceive structurally related signals and differ in downstream kinase signaling. The results of these 4 GmCRK-pairs suggest that each member of a pair could represent neofunctionalization of these highly similar GmCRKs. In the other 4 GmCRK pairs, *GmCRK2/GmCRK6* and *GmCRK9/GmCRK63* from clade I, *GmCRK3/GmCRK25* from clade II, and *GmCRK1/GmCRK62* from clade III, both members of each pair exhibited alternated peaks of maximum transcript levels in specific plant tissues (S4 Fig). For example, for the pair *GmCRK2/GmCRK6*, the expression of *GmCRK2* is only higher than *GmCRK6* in shoot apical meristems, while in the rest of the plant tissues analyzed, both members could co-express but *GmCRK6* exhibit the maximum levels of transcripts. Similarly, both members of the pair *GmCRK1*/*GmCRK62* showed co-expression in most tissues, while *GmCRK1* is highly expressed in shoot apical meristem, and *GmCRK62* is highly expressed in leaves. The members of the pairs *GmCRK9*/*GmCRK63* and *GmCRK3*/*GmCRK25* co-express in most tissues, and each member of a pair alternate the maximum transcript levels in different plant tissues (S4 Fig). Taken together, the results suggest that gene expression of each member of these 8 *GmCRKs* pairs is precisely controlled, and high peaks of expression could represent predominant roles for specific GmCRKs in different plant tissues.

### Expression patterns of *GmCRKs* in response to PAMPs and pathogens

**Fig 3:**
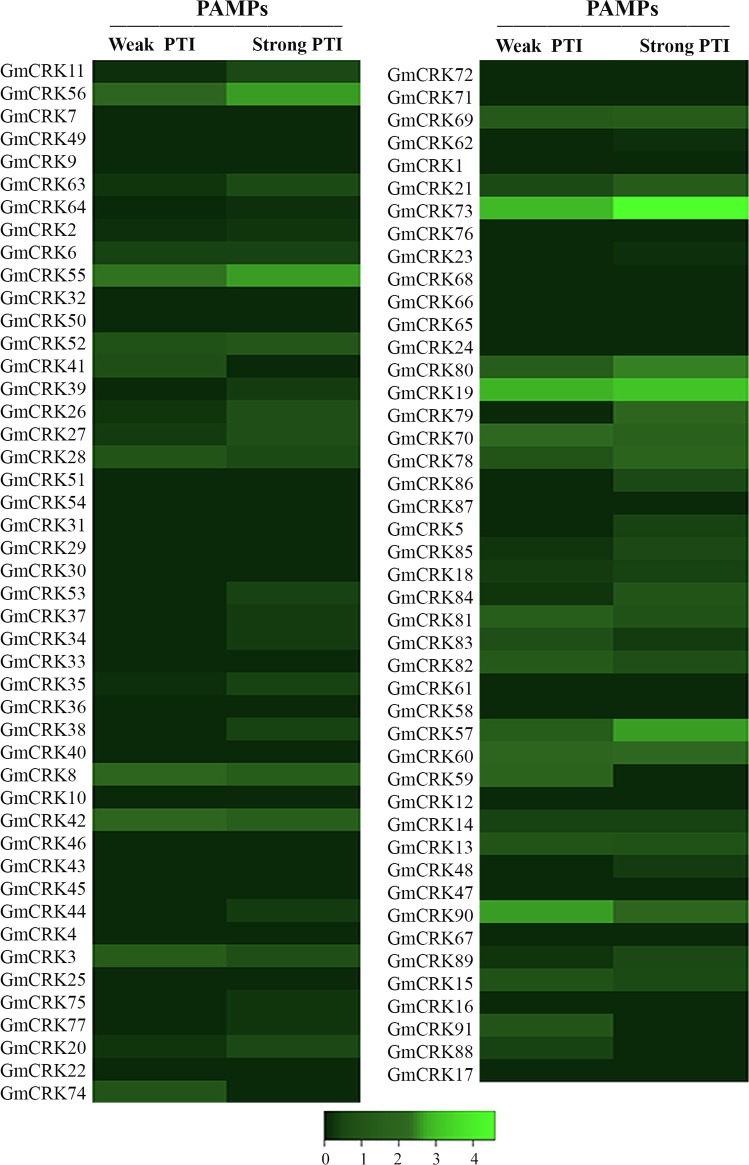
Expression of *GmCRKs* in response to PAMPs. RNA-seq data of flg22+chitin treated tissues. Expression values were retrieved from the 'Genevestigator' database. Expression intensities of PAMPs-treated samples were compared to untreated control experiments and log_2_-ratios were used for visualization. Differences were considered when changes were ≥2-fold. Two genotypes with contrasting responses were used for PAMPs (weak and strong PTI). The resulting heatmap is color coded as illustrated.

To gain further insight into the role played by *GmCRKs* during biotic stress, we analyzed digital expression profiles of tissues exposed to PAMPs (flg22+Chitin), and two important soybean pathogens, including the fungus *Phakopsora pachyrhizi* (*P*. *pachyrhizi*) and the oomycete *Phytophthora sojae* (*P*. *sojae*), using RNA-seq and microarray based expression data available at Genevestigator [39]. PAMPs treatment increased the expression levels (≥ two fold) of 10 of the 91 GmCRK genes (Fig 3; S10 Table). When two contrasting genotypes were compared, LDX01-1-65 with a weak PTI response and LD00-2817 P with a strong PTI response, we could observe that *GmCRK57*, *73*, *79*, and *80*, expressed higher transcript levels in the genotype with a strong PTI response (S10 Table).

**Fig 4:**
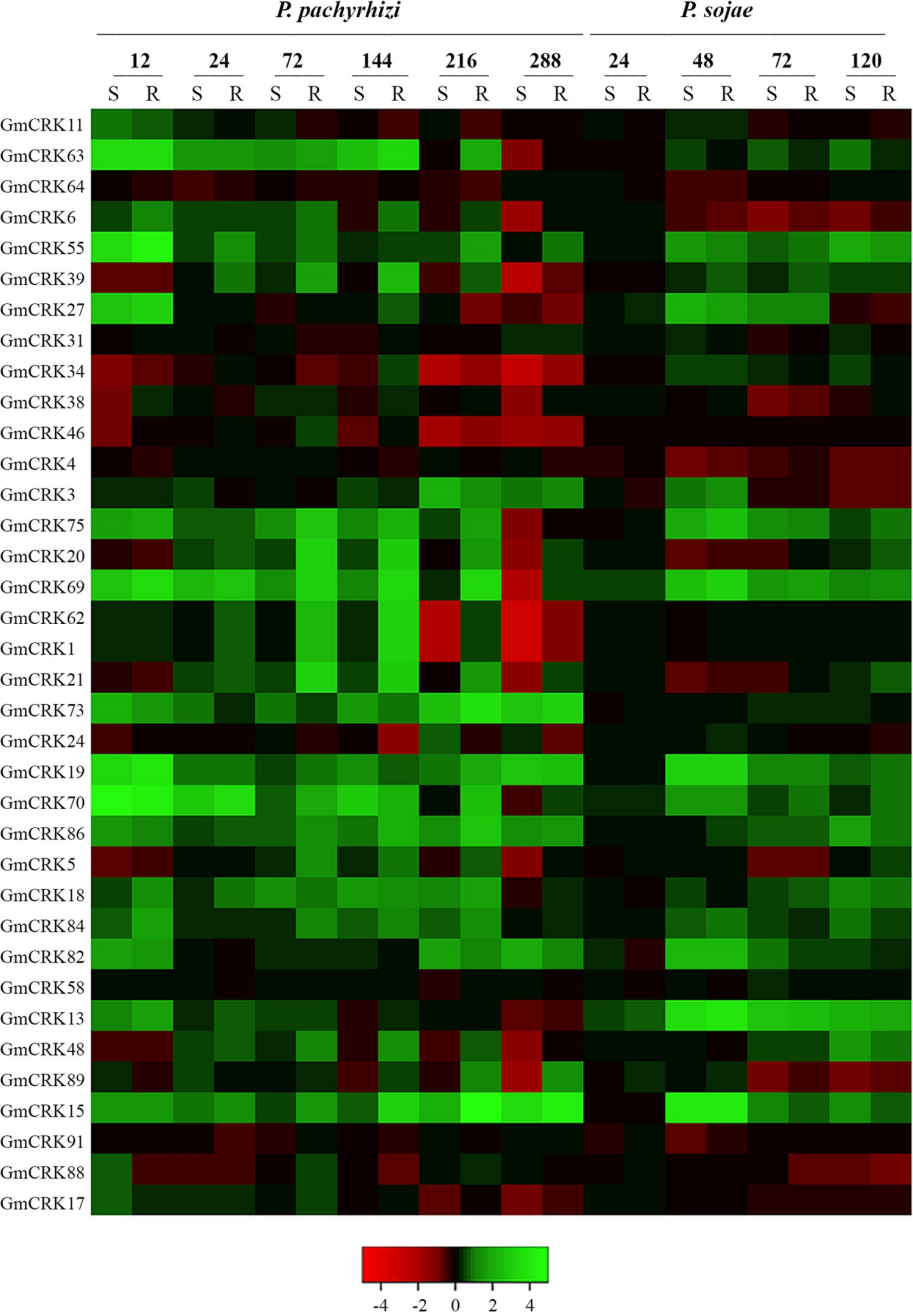
Expression of *GmCRKs* in response to pathogens. Microarray expression data of *Phakopsora pachyrhizi* (*P*. *pachyrhizi*) and *Phytophthora sojae* (*P*. *sojae*) inoculated tissues. Expression values were retrieved from the 'Genevestigator' database. Expression intensities of pathogen-treated samples were compared to untreated control experiments and log_2_-ratios were used for visualization. Differences were considered when changes were ≥2-fold. Two genotypes with contrasting responses were used for both pathogens (resistant (R) and susceptible (S)). The resulting heatmap is color coded as illustrated.

Microarray expression data for pathogen-infected tissues were only available for 36 *GmCRKs* (Fig 4; S10 Table). Only genes that were induced ≥2-fold or repressed ≥2-fold were considered. In response to *P*. *pachyrhizi*, 16 *GmCRKs* were induced at different times post-inoculation. *GmCRKs* exhibited different expression patterns in response to this pathogen. While *GmCRK63*, *69* and *70* are induced at 12 hours post inoculation (hpi) and maintained at most time points until 216 hpi, *GmCRK27* and *GmCRK55* are only induced at 12 hpi, and *GmCRK1*, *20*, *21*, *62*, and *75* are induced later, and only at 72 and 144 hpi. Other *GmCRKs* are induced after 144–216 hpi (*GmCRK15*, *39*, *3*, *73* and *86*). *GmCRK63* is induced at 12 and 144 hpi, and *GmCRK19* at 12 and 288 hpi. Interestingly, several *GmCRKs* were only expressed in the resistant genotype, including *GmCRK1*, *20*, *21*, *39*, *62*, *75*, and *86*, while the expression levels of *GmCRK15* and 69 were significantly higher in the resistant compared to the susceptible genotype. Furthermore, at the latest time points of *P*. *pachyrhizi* inoculation (216 and/or 288 hpi), 5 *GmCRKs* were repressed in the susceptible genotype but not in the resistant genotype (*GmCRK1*, *34*, *39*, *62*, and *69*) (Fig 4; S10 Table). Infection with the oomycete *P*. *sojae* showed less variation in *GmCRKs* expression. *P*. *sojae* treatment induced the expression levels of 7 *GmCRKs* and no *GmCRK* was repressed. Expression levels of 6 *GmCRK* transcripts are only induced at 48 hpi (*GmCRK15*, *19*, *27*, *69*, *75*, *82*), while *GmCRK13* is induced at 48, 72 and 120 hpi. Furthermore, *GmCRKs* expression patterns in response to this oomycete were similar in both genotypes (susceptible and resistant). Taken together, these results show that 10 *GmCRKs* are upregulated with PAMPs, 17 *GmCRKs* exhibited altered expression in plant tissues responding to *P*. *pachyrhizi*, and 7 *GmCRKs* are upregulated with the oomycete *P*. *sojae*. When we focused on the members of the 9 segmental duplication pairs (S5 Table), two members of different pairs, *GmCRK56* and *GmCRK73* resulted induced by PAMPs in both genotypes while none of the corresponding *GmCRK* counterparts, *GmCRK11* and *GmCRK21*, respectively, were induced. Microarray expression data for pathogen-infected tissues were available for three segmental duplication pairs. Members of the pair *GmCRK1/GmCRK62* are both induced at 72 and 144 hpi in the resistant genotype and repressed at 216 and 288 hpi in the susceptible genotype (Fig 4; S10 Table). The pair *GmCRK18/GmCRK84* exhibited in general slight but consistent differences in the transcriptional ratio of the pair between susceptible and resistant genotypes to *P*. *pachyrhizi*. However, the pair *GmCRK21*/*GmCRK73* showed contrasting differences in the expression patterns. The transcriptional profiles evidenced a balanced expression of *GmCRK21*/*GmCRK73*, and each member alternate in maximum transcript levels at different time points of leaves of the same cultivar responding to *P*. *pachyrhizi*, changing the transcriptional ratio of the pair during the plant defense response in each genotype (Fig 4). Furthermore, both members of the pair *GmCRK21/GmCRK73* exhibit a different transcriptional ratio between resistant and susceptible genotypes responding to *P*. *pachyrhizi*. These results suggest a precise control of the transcriptional ratio of the pair GmCRK21/GmCRK73, which differ at specific time points in susceptible and resistant genotypes to fungal pathogens. We also observed in more detail the expression levels of *GmCRKs* with 4 modules DUF26 in response to PAMPs. Three *GmCRKs* were induced with PAMPs, including *GmCRK57*, *60*, and *90*. In addition, microarray expression profiles showed that *GmCRK48* is not induced with pathogens, while *GmCRK13* is induced in all time points with *P*. *sojae*. Taken together, the transcript expression profiles of *GmCRKs* indicate an active role of several members of the family involved in plant defense to biotic stress, and suggest that some of them could play important roles associated to activation of plant immunity.

### Oxidative burst in response to PAMPs and DAMPs

**Fig 5:**
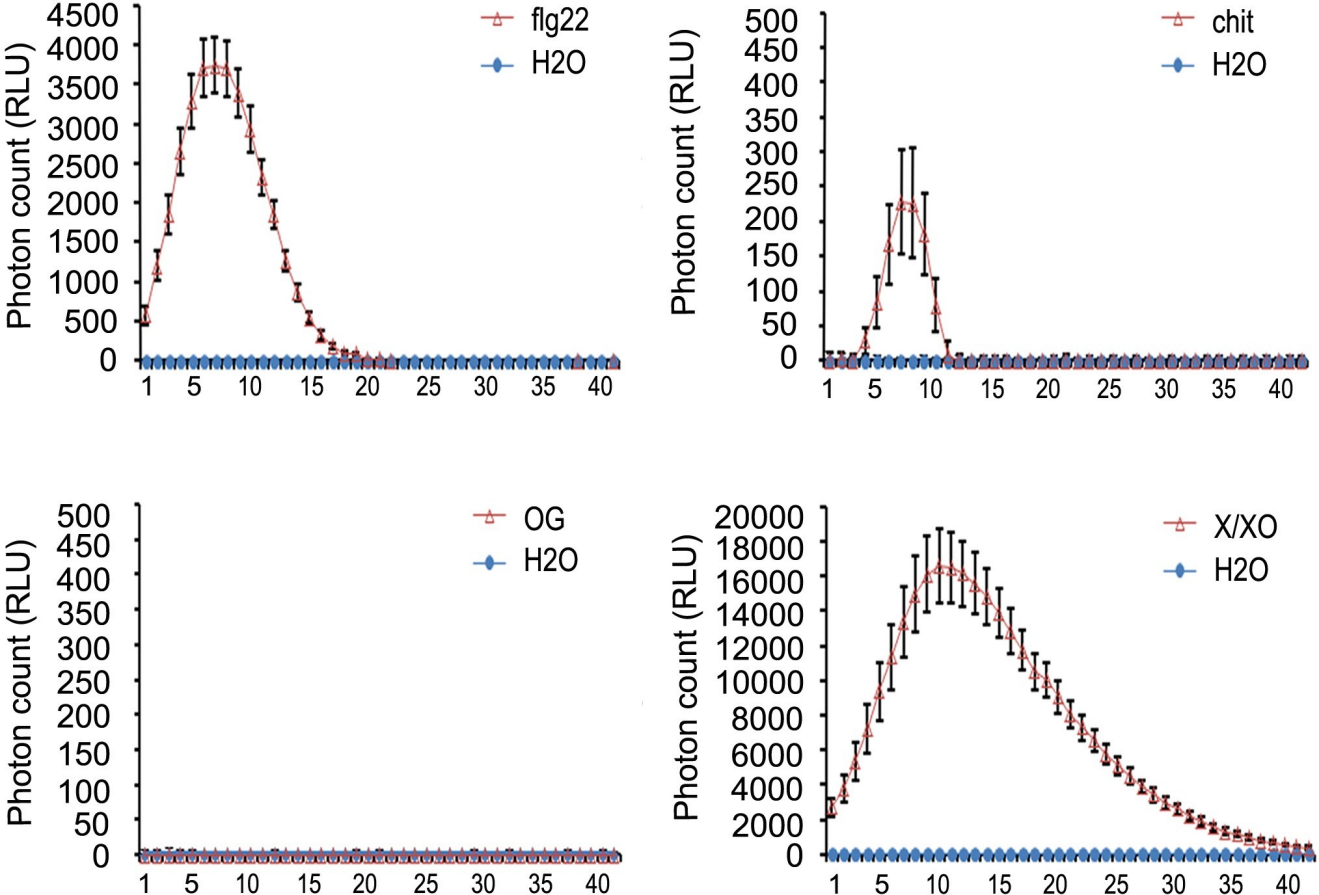
Oxidative burst triggered by flg22, chitin, OG and xanthine/xanthine oxidase (X/XO). Flg22-, chitin-, OG-induced ROS bursts were measured in relative luminescence units (RLU). Values are mean ±SE (n = 18). X/XO treatment was used as control. Time kinetics of the MAMP triggered oxidative burst are shown. Experiments were repeated three times with similar results.

Recognition of PAMPs by plant cells induces rapidly the production of ROS [2], and considering the DUF26 structural characteristics, it has been hypothesized that CRKs could act as components of ROS sensory systems [15]. Therefore, we decided to measure ROS production as an early PTI response to PAMPs (flg22 and chitin) in soybean tissues (Fig 5). Since OG fragments act as DAMPs, and in potato short OG (dimers and trimmers of polygalacturonic acid) induce the expression of CRKs [25], we also measured ROS production in response to OG. In this work, we have used the enzymatic system Xanthine-Xanthine Oxidase (X/XO) in plant treatments as an external ROS signal uncoupled to PAMPs, therefore we measure the intensity of such ROS signal in plant treatments. As expected, the amplitude and wavelength of ROS generated by the X/XO treatment was significantly higher than the one observed in PAMPs-treated samples, and it is mainly provided by the X/XO ROS-generation system. Nevertheless, these measurements provide an estimation of the magnitude and time extent of ROS during plant treatments. Short OGs did not trigger an oxidative burst in soybean tissues, which is consistent with previously reported data [48]. Both PAMPs, flg22 and chitin, triggered an oxidative burst, and ROS production exhibited higher amplitude and a longer wavelength in flg22 treatment compared to chitin. These results indicate that each PAMP, either flg22 or chitin, trigger different types of ROS waves with specific characteristics.

### Expression patterns of selected *GmCRKs* in response to MAMPs, DAMPs and ROS

**Fig 6:**
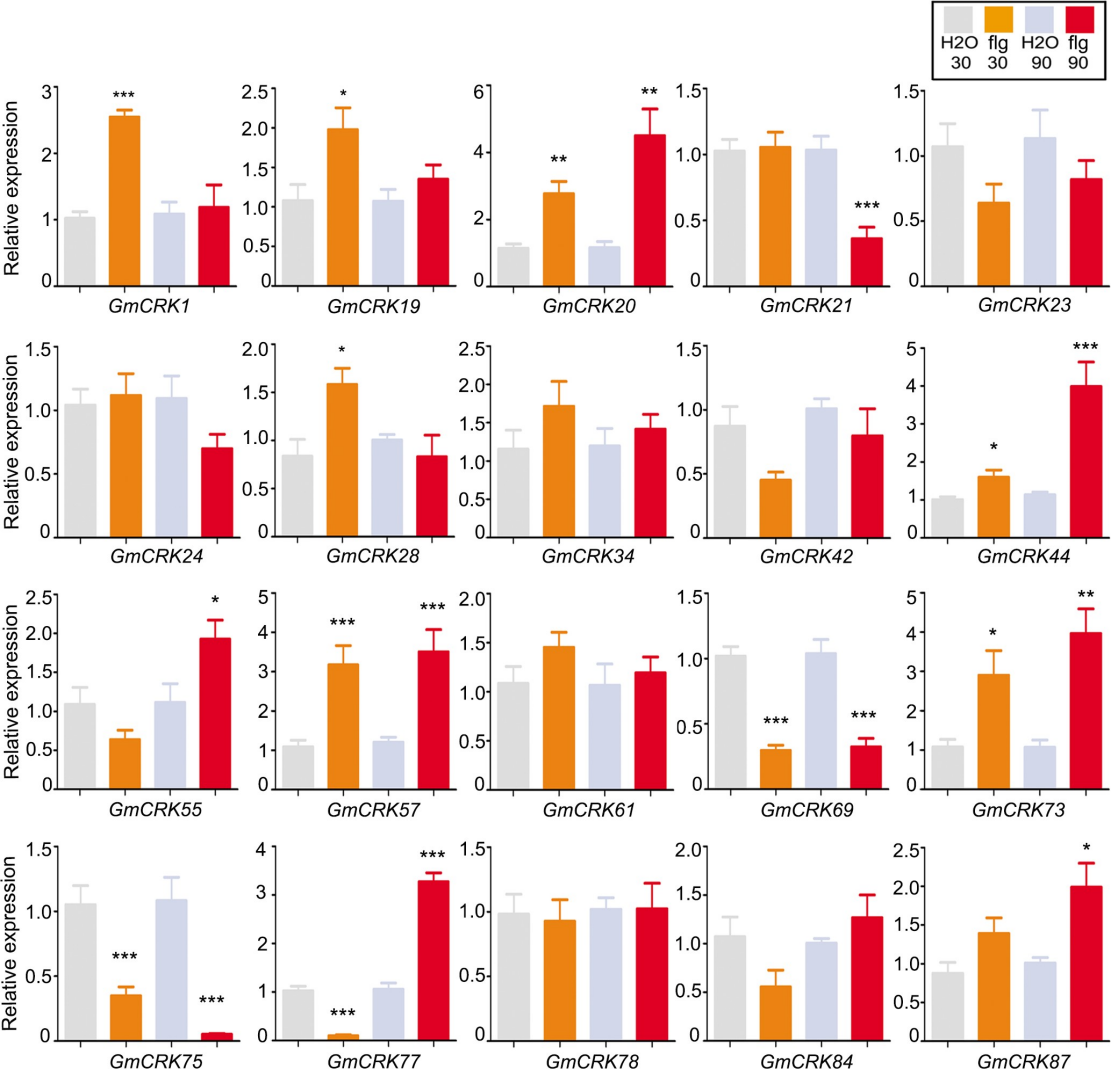
*GmCRKs* expression levels in response to flg22. RT-qPCR analysis of selected CRK genes at 30 and 90 min after flg22-treatment. EF1α gene was used as the reference gene. The expression levels in flg22-treated tissues are relative to the corresponding level of expression in water-treated tissues at the indicated time points. Results are reported as means ± SE of three biological replicates. Asterisks indicate a statistically significant difference between flg22-treated and water-treated tissues (Students t-test, *P < 0.05; **P < 0.01; ***P < 0.001). Biological significance was considered when differences in expression values were ≥ 2 fold.

Based on protein structure and phylogenetic position, 20 genes were selected to perform further expression analyses in response to PAMPs, DAMPs, and external ROS signals. To select the *GmCRKs* for such analysis we have considered the following criteria: (i) genes should be distributed in all clades; (ii) at least some genes with ectodomains containing 4 modules of DUF26 should be included; (iii) to explore deeper the expression of the GmCRK family, a subgroup of genes from clade III was selected. To analyze *GmCRKs* mRNA accumulation during plant immunity, flg22 and chitin were used as PAMPs, short OG as a DAMP, and X/XO treatment was used as an external ROS signal uncoupled to PAMPs. Flg22 treatment induced the expression levels of 9 *GmCRKs* (≥ 2 fold), which are distributed in the main four clades (Fig 6). Two *GmCRKs* are only induced at 30 min (*GmCRK1* and *GmCRK19*), four are only induced at 90 min (*GmCRK44*, *GmCRK55*, *GmCRK77* and *GmCRK87*), and three are induced at both time points (*GmCRK20*, *GmCRK73* and *GmCRK57*). Flg22 also triggers *GmCRK* repression (≥ 2 fold). *GmCRK69* and *GmCRK75* are repressed at 30 and 90 min post treatment, *GmCRK77* is only repressed at 30 min and *GmCRK21* is only repressed at 90 min.

**Fig 7:**
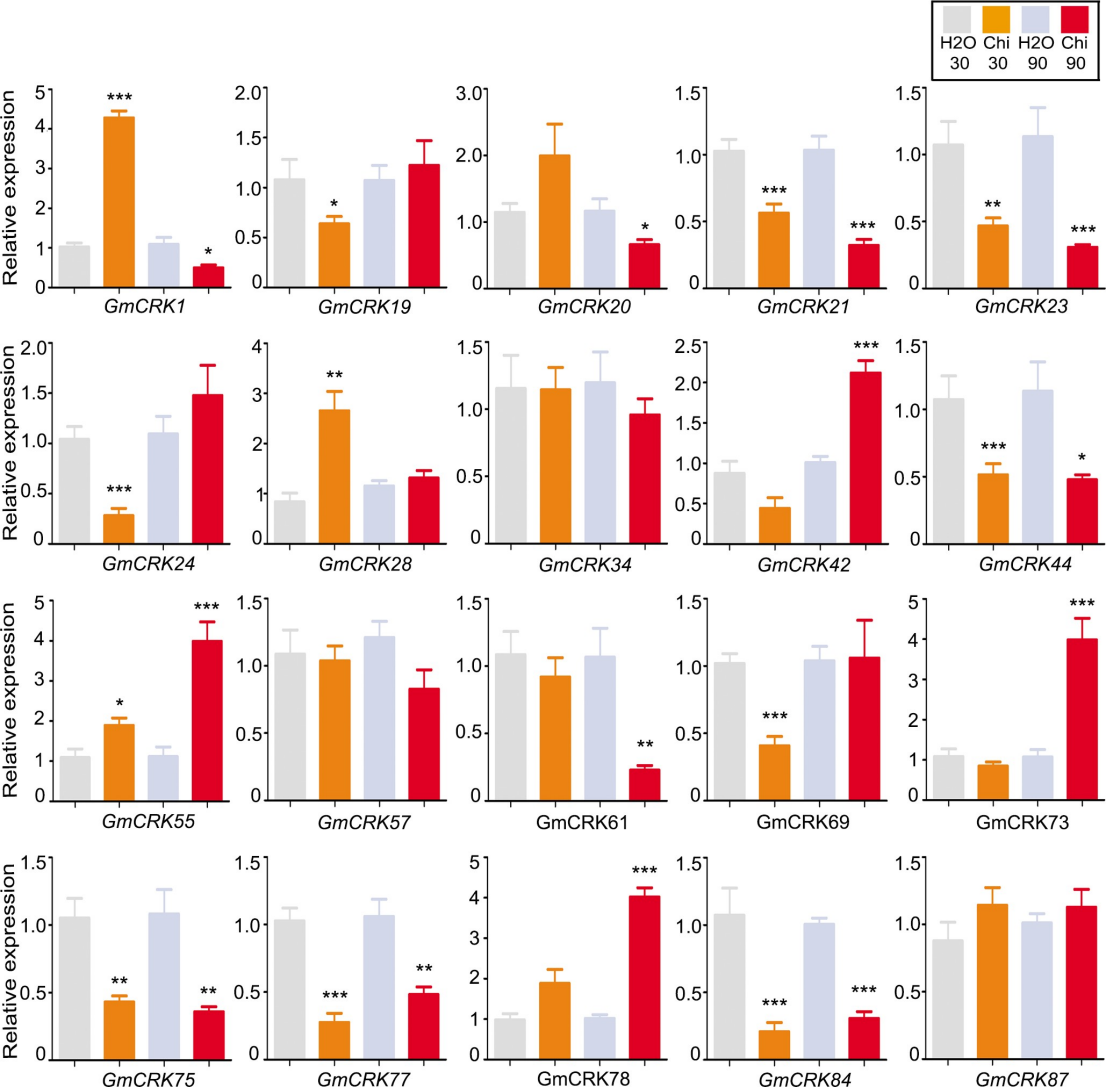
*GmCRKs* expression levels in response to chitin. RT-qPCR analysis of selected CRK genes at 30 and 90 min after chitin (Ch)-treatment. EF1α gene was used as the reference gene. The expression levels in chitin-treated tissues are relative to the corresponding level of expression in water-treated tissues at the indicated time points. Results are reported as means ± SE of three biological replicates. Asterisks indicate a statistically significant difference between chitin-treated and water-treated tissues (Students t-test, *P < 0.05; **P < 0.01; ***P < 0.001). Biological significance was considered when differences in expression values were ≥ 2 fold.

Chitin induced the expression levels of six *GmCRKs* that are distributed in clade I, II and III (Fig 7). Two *GmCRKs* are induced by chitin at 30 min (*GmCRK1* and *GmCRK28*), three at 90 min (*GmCRK42*, *GmCRK73* and *GmCRK78*), and *GmCRK55* was induced at 30 and 90 min. Chitin also repressed the expression of 10 *GmCRKs*, two *GmCRKs* at 30 min (*GmCRK24* and *GmCRK69*), two *GmCRKs* at 90 min (*GmCRK1* and *GmCRK61*), and 6 *GmCRKs* were repressed at both time points (*GmCRK21*, *GmCRK23*, *GmCRK44*, *GmCRK75*, *GmCRK77*, and *GmCRK84*).

**Fig 8:**
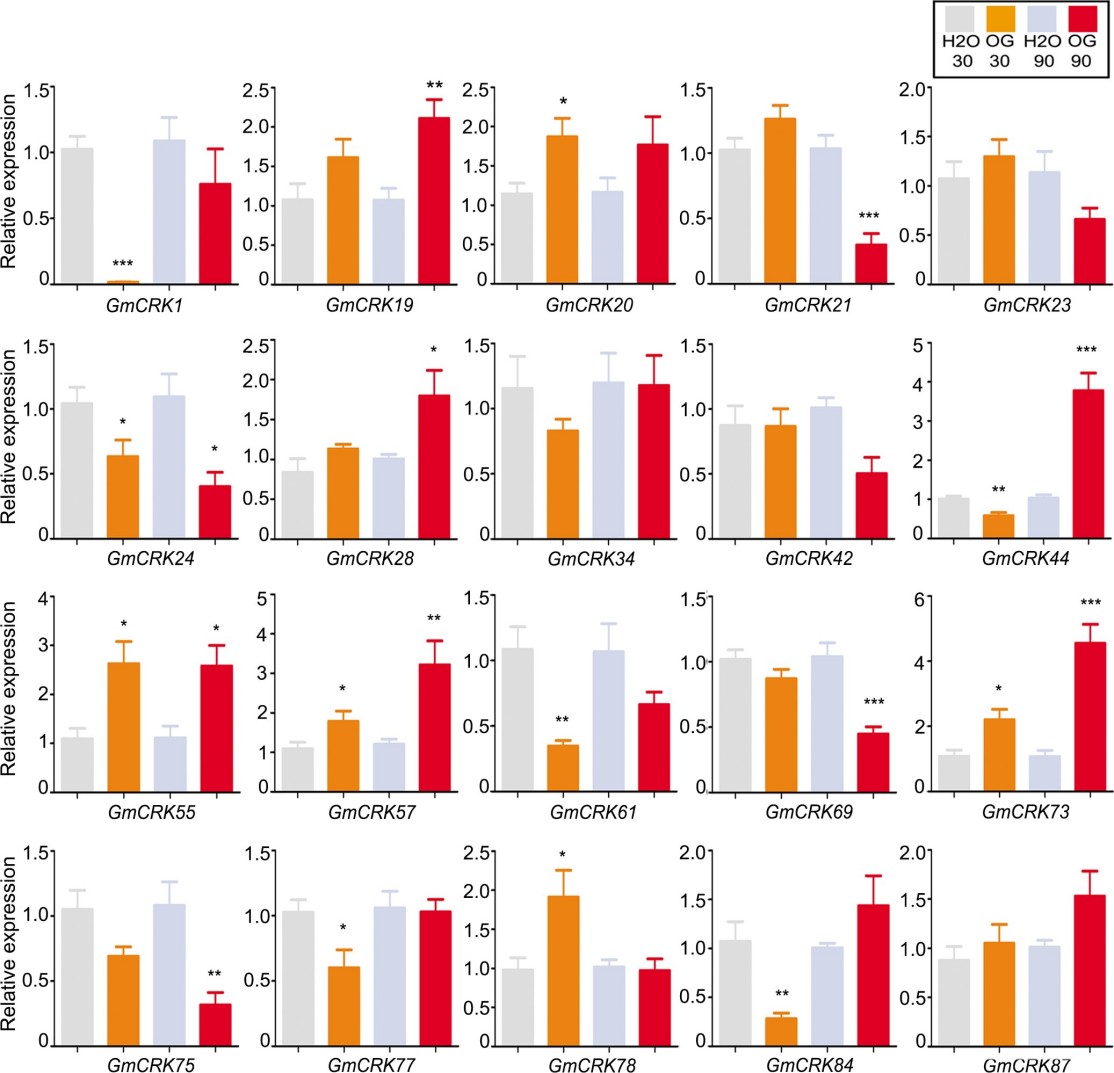
*GmCRKs* expression levels in response to oligogalacturonides (OG). RT-qPCR analysis of selected CRK genes at 30 and 90 min after oligogalacturonides (OG)-treatment. EF1α gene was used as the reference gene. The expression levels in OG-treated tissues are relative to the corresponding level of expression in water-treated tissues at the indicated time points. Results are reported as means ± SE of three biological replicates. Asterisks indicate a statistically significant difference between OG-treated and water-treated tissues (Students t-test, *P < 0.05; **P < 0.01; ***P < 0.001). Biological significance was considered when differences in expression values were ≥ 2 fold.

OG treatment induced the expression of 8 GmCRK-encoding genes, which are distributed in the main four clades (Fig 8). Two *GmCRKs* are induced at 30 min (*GmCRK20* and *GmCRK78*), three at 90 min (*GmCRK19*, *GmCRK28*, *GmCRK44*), and three *GmCRKs* are induced at 30 and 90 min (*GmCRK55*, *GmCRK57*, *GmCRK73*). Repression of *GmCRKs* by OG was observed for *GmCRK1*, *GmCRK44*, *GmCRK61*, and *GmCRK84* at 30 min, and *GmCRK21*, *GmCRK69*, and *GmCRK75* at 90 min), while *GmCRK24* was repressed at both time points.

**Fig 9:**
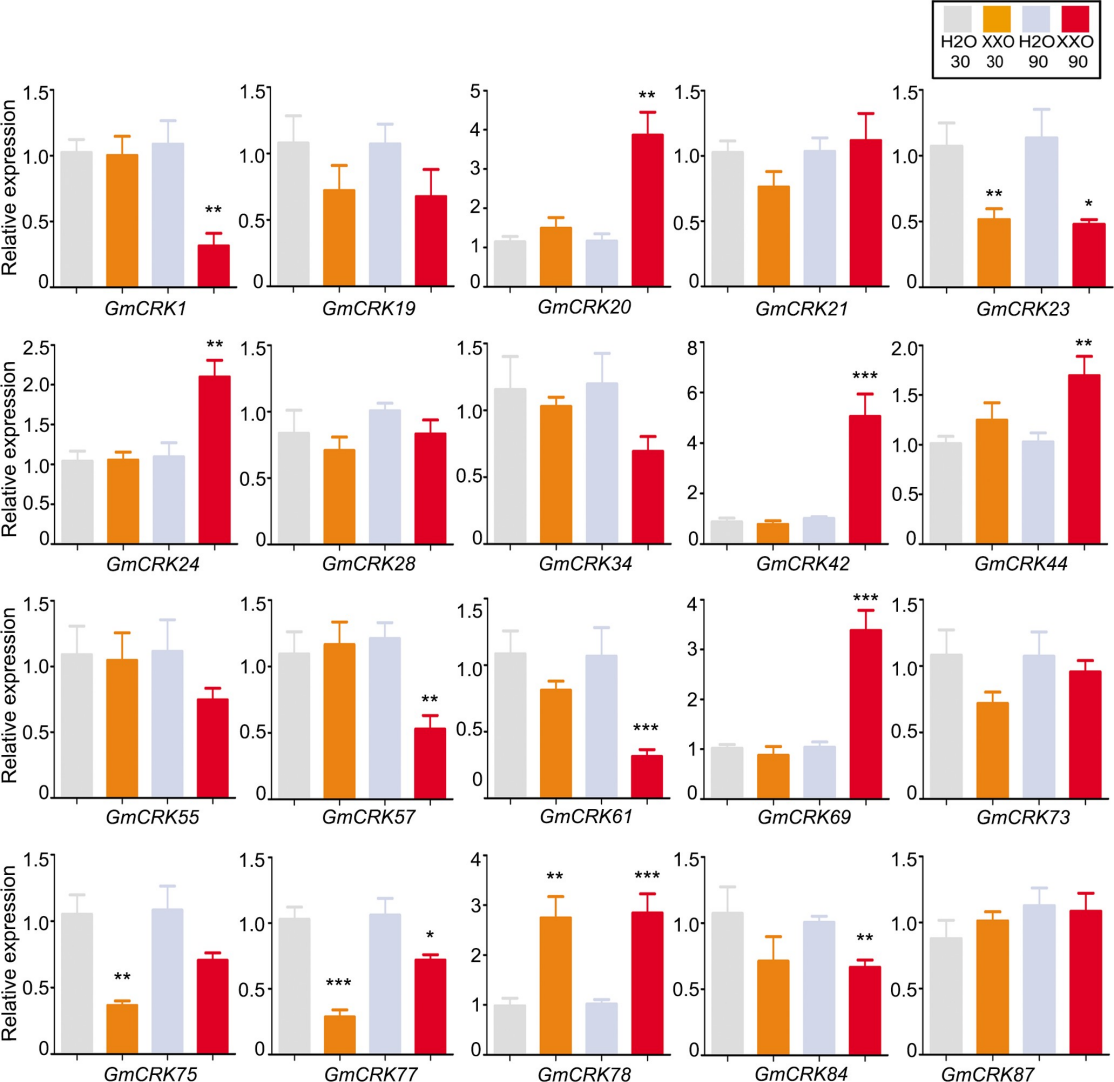
*GmCRKs* expression levels in response to xanthine/xanthine oxidase (X/XO). RT-qPCR analysis of selected CRK genes at 30 and 90 min after xanthine/xanthine oxidase (X/XO)-treatment. EF1α gene was used as the reference gene. The expression levels in X/XO-treated tissues are relative to the corresponding level of expression in water-treated tissues at the indicated time points. Results are reported as means ± SE of three biological replicates. Asterisks indicate a statistically significant difference between X/XO-treated and water-treated tissues (Students t-test, *P < 0.05; **P < 0.01; ***P < 0.001). Biological significance was considered when differences in expression values were ≥ 2 fold.

X/XO treatment induced the expression of 5 *GmCRKs* from clade II and III (Fig 9). *GmCRK20*, *GmCRK24*, *GmCRK42*, and *GmCRK69* were induced by X/XO at 90 min, and *GmCRK78* was induced at both time points. X/XO also repressed the expression of *GmCRK75* and *GmCRK77* at 30 min, and *GmCRK1*, *GmCRK57* and *GmCRK61* at 90 min, while *GmCRK23* was repressed at both time points. Interestingly, several *GmCRKs* (*GmCRK19*, *GmCRK28*, *GmCRK87*) induced by Flg22, chitin or OG, did not respond to X/XO treatment. Similarly, the segmental pair *GmCRK21*/ *GmCRK73* exhibited modulated expression by PAMPs and OG, but they did not respond to X/XO treatment. In contrast, *GmCRK24* and *GmCRK69* induced by X/XO, are repressed or remain at control levels in PAMPs and OG treatments, while *GmCRK57* was repressed by X/XO, induced by Flg22 and OG, and exhibited control levels for chitin. These results indicated that external ROS signals produced by X/XO induced different *GmCRKs* expression patterns than PAMPs that trigger ROS production and OG treatments that do not produce ROS. The results suggest that the transcriptional control of *GmCRKs* is very sensitive to external stimuli and display signal-specific expression patterns.

Several *GmCRK* exhibit dual regulation during the 90 min period post treatment with flg22, chitin, or OG. *GmCRK77* is significantly repressed at 30 min post flg22 treatment (≥3 fold), while it is highly induced at 90 min (≥3 fold). Similarly, *GmCRK44* is repressed at 30 min of OG treatment (≥2 fold), while it is highly induced at 90 min (≥4 fold). *GmCRK1* is highly induced at 30 min of chitin treatment (≥4 fold), while it is repressed at 90 min (≥2 fold). These results suggest that the expression of these *GmCRKs* is precisely controlled, probably by continuous modulation of transcriptional profiles influenced by constant perception of intra and extracellular stimuli. Interestingly, members of the segmental pair *GmCRK21*/*GmCRK73* showed different expression patterns. In contrast to *GmCRK21*, which was repressed by flg22, chitin and OG treatments, *GmCRK73* was induced with all these treatments. These results suggest that the members of the pair *GmCRK21*/*GmCRK73* exhibit antagonist transcriptional regulation. Taken together, the results show that all the 20 *GmCRKs* analyzed by RT-qPCR exhibited differential expression patterns in response to flg22, chitin or OG, compared to control treatment, except for *GmCRK34* that in all treatments exhibited transcript levels similar to control samples. Some *GmCRKs* are responsive to all treatments, including *GmCRK55* and *GmCRK73* (induced), and *GmCRK21*, *GmCRK69*, and *GmCRK75* (repressed), suggesting that these GmCRKs could play primary roles in soybean leaves responding to biotic stress signals. The results suggest a fine-tuning control of *GmCRK* transcriptional regulation adjusted by continuous perception of intra and extracellular stimuli.

### Analysis of CRKs from other crops

The presence of 11 GmCRKs with ectodomains containing 4 modules of DUF26 domains in *G*. *max* prompted us to explore whether CRKs from other crops exhibit this type of CRK receptors. Therefore, we explored NCBI and Phytozome databases using as query GmCRK12. We found that several crops including fruit trees, cotton, and plants of the Fabaceae family exhibited CRKs of this type (S2 Table). These results indicate that CRKs with ectodomains containing 4 modules of DUF26 are present in other crops, including the legumes *M*. *truncatula* and *P*. *vulgaris*. Subsequently, we have explored the Phytozome database searching for all CRKs from *M*. *truncatula* and *P*. *vulgaris*, and retrieved and analyzed all the sequences (S3 Table; S5 Fig). In *M*. *truncatula* we found 53 MtCRKs, 43 with 2 modules of DUF26 domains within the ectodomain, seven with 4 modules, while a few exhibited one or three modules. In *P*. *vulgaris* we found 54 PvCRKs, 50 exhibited two modules of DUF26 domains within the ectodomain, two with 4 modules, and a few with one or three modules. These results indicate that *M*. *truncatula* and *P*. *vulgaris* exhibit a modular organization of CRKs ectodomains similar to *G*. *max*. Surprisingly, the ectodomains of MtCRKs and PvCRKs containing 4 modules of DUF26 domain are all encoded by only one exon similar as those in *G*. *max* (S1 and S5 Figs). These results suggest that during plant evolution similar mechanisms have expanded the repertoire of CRKs within these closely related plants of the Fabaceae family. We have also analyzed the expression of these type of CRKs in *M*. *truncatula*, *P*. *vulgaris*, and *G*. *max* exploring the Phytozome database (S6 Fig). The results indicated that in these plants several CRKs containing 4 modules of DUF26 within the ectodomain, are expressed in several plant tissues under diverse experimental conditions, including roots and leaves under symbiotic conditions (S6 Fig; S11 Table). Taken together these results suggest that this type of CRKs is involved in perception of external stimuli, and they could play different and novel specific functions perceiving apoplastic redox status and stimuli within plant species.

## Discussion

Plant perception of biotic stress signals mediated by PRR-RLKs is central to activate plant immunity. Biotic stress signals such as PAMPs trigger the integration of immune receptor complexes, signaling plant immune responses [1]. Early immune signaling responses include Ca^2+^ influx, ROS production, kinase signaling and transcriptional gene regulation [2]. Surface localized CRKs have been implicated in *Arabidopsis* plant immunity [21], and they have been proposed as possible candidates involved in ROS perception [15, 17]. In this study, we performed a genome-wide analysis of soybean (*G*. *max*), and identified 91 *GmCRKs*. Two whole genome duplication events took place twice in the palaeopolyploid genome of *G*. *max* in 59 and 13 million years ago, respectively [22]. Segmental and tandem duplications events influenced the expansion of several gene families, including the RLK family [7]. Consistently, we found that 81% of the *GmCRKs* integrate tandem repeats, and 24 segmental duplication events were identified. Previous reports indicated that chromosome 20 is highly homologous to the long arm of chromosome 10 [22]. Consistently we found duplication events of GmCRKs in the long arm of chromosome 10 and chromosome 20. We identified 18 groups of *GmCRKs* located in tandem repeats, and 9 pairs of GmCRKs representing segmental duplications of the family that exhibit high protein similarity (91–98%). Consistently, it has been previously suggested that tandem duplications and chromosomal duplication could have contribute to the expansion of the *AtCRK* gene family in *Arabidopsis* [6]. Furthermore, previous reports showed that plant RLKs containing ectodomains with DUF26 exhibit high rates of tandem duplication [49]. Although similar mechanisms seem to be involved in CRK expansion of angiosperms, their evolution seems specific for each plant species. The high number of soybean GmCRKs nearly doubles the number present in *Arabidopsis* and rice families [14,16,50]. Like *Arabidopsis* and rice CRK families, the ectodomain structure of most of the GmCRKs exhibit a bi-modular organization, each module containing one DUF26 domain, and a few GmCRKs only exhibited one module. Interestingly, the ectodomain of 11 GmCRKs, distributed in 4 chromosomes, exhibit a duplicated bi-modular organization of the ectodomains, each containing 4 DUF26 domains. Phylogenetic and genome analysis of these 11 GmCRKs suggest that they have been generated by segmental and tandem duplication events, and different transcriptional profiles in soybean tissues suggest that they are functional and could play novel functions. All GmCRKs with an ectodomain containing 4 modules of DUF26 domains are encoded by only one exon, suggesting that they could have derived from duplication events of *GmCRKs* genes with two modules of DUF26 encoded by a single exon. Thus, these 11 GmCRKs represent a structural duplication of the ectodomain involved in perception of signals developed during soybean evolution. Considering the structural features of the ectodomains of the *Arabidospsis* and rice CRK families, and those of the soybean GmCRK family, the presence of 11 GmCRKs with ectodomains containing 4 DUF26 indicate that the CRK family evolved differently in these plants, most likely by specific selective pressure on ectodomains involved in plant perception of environmental stimuli. However, our comparative analysis of CRKs organization within the Fabaceae family suggests that similar molecular mechanisms drove evolution within this family, especially by duplication of bi-modular organization of DUF26 domains. Therefore, the evolution of plants CRKs seems to expand perception of the apoplast by amino acid sequence diversity and different modular organization of DUF26 domains within the ectodomain of CRKs.

The analysis of publicly available expression data for Gm*CRK*s in soybean indicated that several members exhibited distinct expression patterns in tissues/organs. Nearly one third of the *GmCRKs* are expressed in more than three types of tissue, 30 to 50 *GmCRKs* are expressed in leaves, nodules, roots, flowers, and stems. Some *GmCRKs* are highly expressed in a tissue-specific mode, suggesting tissue-specific roles for some member of the family. Furthermore, analysis of contrasting soybean phenotypes for weak and strong immune response, indicated that (flg22+chitin)-treated plants exhibited differential gene regulation for *GmCRKs*. Similarly, data obtained from pathogen-treated soybean suggested that *GmCRKs* transcriptional regulation is genotype specific, and pathogen specific. Interestingly, in response to *P*. *pachyrhizi* several *GmCRKs* were only expressed in the resistant genotype, while the expression levels of other *GmCRKs* were significantly higher in the resistant than in the susceptible genotype. Furthermore, several *GmCRKs* were repressed at late time points after infection in the susceptible genotype but not in the resistant genotype, suggesting that GmCRKs could play important roles in plant immunity. The role of *CRKs* related to plant defense responses to phytopathogens has been identified in several plant species including potato [25], barley [51], rice [52], and *Arabidopsis* [18,53,54].

The analysis of the 9 selected segmental GmCRK-pairs, each pair sharing high sequence similarity (>91%), revealed different modes of transcriptional regulation. For example, the pair *GmCRK16/GmCRK91* mostly exhibited similar regulation of each member of the pair, and they seem to signal through different kinase domains, suggesting that they could function cooperatively, by perceiving structurally related signals, and amplifying such signals through different downstream kinase components. However, each of the other 8 segmental pairs identified exhibited antagonistic modes of regulation for each member of a pair in plants treatments, suggesting that the balanced ratio of transcripts of the pair could be important for the role played by these GmCRKs. Our transcriptional analysis confirmed that the receptor pair *GmCRK21*/*GmCRK73* exhibited an antagonistic mode of regulation in different plant treatments, while *GmCRK21* was downregulated by flg22, chitin, and OG, *GmCRK73* transcript levels increased with all three treatments. Antagonistic modes of regulation of these GmCRK pairs exhibiting high protein similarity suggest that each member of a pair could play novel specific functions. Accordingly, the GmCRKs members of each pair exhibited differences in their promoter elements, suggesting that each member of a segmental pair could play specific roles in cells responding to environmental stimuli. For example, while both *GmCRK73* and *GmCRK21* have cis-regulatory elements related to methyl jasmonate, defense and stress responsiveness in their promoters, only *GmCRK21* has a salicylic acid and a heat responsive element, and only *GmCRK73* has a gibberellin and a flavonoid related element. Consistently, transcriptional regulation of *AtCRKs* in *Arabidopsis* has been shown to be controlled by multiple plant signaling components, including methyl jasmonate, ethylene, salicylic acid, and ROS [16,55]. Large-scale phenomics analysis of *Arabidopsis crk* mutants suggested primary and fine-tuned roles for members of this family related to oxidative stress [17]. The role of some specific CRKs have been related to program cell death [18,21,55], regulation of plant growth, and ABA signaling [19,20]. Therefore, plant CRKs emerged as a family related to perception of environmental stimuli related to abiotic and biotic stress signals. The role of CRK ectodomains is still unknown, and to our knowledge, ligands for plant CRKs remain to be found. The plant CRK-ectodomains based on DUF26 modular organization hint such domains as an important structural feature of the family. The structural characteristic of ginkbilobin-2 (Gnk2), a protein with suggested antimicrobial properties from *Ginkgo biloba*, which contain one DUF26 domain, suggest that cysteine form disulphide bonds [56,57]. It has been suggested that the DUF26 structure of plant CRK ectodomains could be target for redox modification leading to structural changes mediated by disulphide bonds [15]. In *Arabidopsis*, DUF26 cysteine residues of the AtCRK28 ectodomain have been shown to be important to trigger plant cell death [21], supporting the hypothesis that DUF26 structure is relevant for the functionality of plant CRKs. ROS production function as a signal amplification system communicating diverse stimuli to nearby cells and systemic tissues [58,59]. Different PAMP signals such as flagellin and chitin, trigger specific ROS waves. Consistently, we have observed specific ROS waves triggered by these PAMPs in soybean tissues, and distinct characteristic patterns of *GmCRK* expression were observed in PAMP- and ROS-treated soybean leaves, suggesting that *GmCRKs* are very sensitive to these signals and respond by displaying signal-specific transcriptional profiles. Furthermore, several of the selected *GmCRKs* analyzed respond to OG, which did not produce a ROS burst, indicating that *GmCRKs* are very sensitive to specific signals even in the absence of an apoplastic ROS burst. Interestingly, AtCRK28 associated with FLS2 in *Arabidopsis*, form a receptor complex integrated by different RLKs triggering plant immunity [21]. *Arabidopsis* lines overexpressing AtCRKs exhibited differences with control plants in ROS production triggered by flg22 [21,54]. Thus, the plant CRKs emerge as a protein family very sensitive to apoplastic stimuli, possibly sensing the redox conditions as well as contributing to the characteristics of specific ROS waves generated by different PAMP signals.

The physicochemical properties of plant CRKs, based on ectodomain sequence variations containing DUF26 domains linked to kinase signaling, as well as the CRKs expanded number, represent a foundation to the functional divergence of perception mechanisms of plant cells. Future biochemical and structural analysis of plant CRKs are needed to find their putative ligands. The analysis of CRKs integrating receptor complexes with other RLKs could partially unmask the role of this family in perception of specific signals. CRKs seem to play a role contributing to the signal amplification by ROS waves generated in plants by specific PAMPs. Analysis of contrasting plant genotypes will contribute to reveal the roles of CRKs in plants responding to environmental stimuli. Finally, such studies will contribute to understand how plants sense the environment and signal the activation of highly specific defense responses as plant immunity.

## Supporting information

S1 Fig The exon/intron structures of *GmCRK* genes based on the phylogenetic tree. Lengths of introns and exons of *GmCRKs* genes are proportionally displayed based on the kilobase scale at the bottom of the figure. Yellow circles indicate GmCRKs with ectodomains containing 4 modules of DUF26.
(TIF)

S2 Fig Gene Ontology (GO) enrichment analysis of GmCRKs. Statistically significant Biological Process GO terms were displayed with AgriGOv2.0. The information inside the boxes includes: GO term, adjusted p-value, GO description, item number mapping the GO in the query list and background, and total number of query list and background.
(TIF)

S3 Fig Expression patterns of *GmCRKs* in soybean organs/tissues. RNA-seq data was retrieved from Phytozome v12.1 and the fragments per kilobase of transcript per million mapped reads (FPKM) value of leaves (L), stem (ST), root (R), root hairs (RT), shoot apical meristem (SAM), nodules (N), flower (F), pod (P) and seed (S) were analyzed. The colored scale bar indicates gene expression level.
(TIF)

S4 Fig Expression pattern of segmental duplicated *GmCRK* pairs in organs/tissues. For each pair, the fragments per kilobase of transcript per million mapped reads (FPKM) values are presented as a line graph.
(TIF)

S5 Fig Domains of legumes CRKs and exon/intron structures of CRKs containing 4 modules of DUF26 domains. **(A)** Pfam domains present in legumes CRK predicted proteins. **(B)** Exon/intron structures of *CRKs* from *Medicago truncatula* and *Phaseolus vulgaris*, each one containing 4 modules of DUF26 within the ectodomains.
(TIF)

S6 Fig Expression pattern of legumes genes encoding CRKs with four DUF26 modules in different organs/tissues/treatments. **(A)** *Glycine max* (Gm), **(B)** *Medicago truncatula* (Medtr), and **(C)** *Phaseolus vulgaris* (Phvu). RNA-seq data were collected from Phytozome v12.1 and fragments per kilobase of transcript per million mapped reads (FPKM) values of leaves (L), stem (ST), root (R), root hairs (RT), shoot apical meristem (SAM), nodules (N), flower (F), pod (P), seed (S), flower open (FO), flower unopen (FU), lateral root standard (LRSt), leaf ammonia (LA), leaf nitrate (LN), leaf standard (LSt), leaf symbiotic condition (LSC), leaf urea (LU), nodules symbiotic condition (NSC), root tip standard (RTSt), root ammonia (RA), root nitrate (RN), root standard (RSt), root symbiotic condition (RSC), root urea (RU), shoot tip standard (SHTSt), stem standard (SSt), flower buds (Fb), green mature pods (GMP), young pods (YP), young trifoliates (YT) are shown. Different colored scale bars for *G*. *max*, *M*. *truncatula* and *P*. *vulgaris* are shown, indicating gene expression level.
(TIF)

S1 Table *GmCRKs* information. (XLS)

S2 Table Example of CRKs containing 4 DUF26 modules in different plant species. (XLS)

S3 Table Locus names, location in the genome, and number of domains of *Medicago truncatula* and *Phaseolus vulgaris* CRK family. (XLS)

S4 Table Primer information. (PDF)

S5 Table Similarities between GmCRK segmental duplicated pairs on the terminal node of the phylogenetic tree. (XLS)

S6 Table Distribution of *GmCRKs* located in tandem along the soybean chromosomes. (XLS)

S7 Table Potential cis-acting regulatory elements in the *GmCRK* promoters. (PDF)

S8 Table List of the cis-regulatory elements identified upstream of the soybean *CRKs*. (XLSX)

S9 Table Expression pattern of *GmCRKs* in different soybean organs/tissues. RNA-seq data were collected from Phytozome v12.1 and fragments per kilobase of transcript per million mapped reads (FPKM) values are shown.
(XLS)

S10 Table Expression pattern of *GmCRKs* in response to MAMPs (flg22+chitin), *Phakopsora pachyrhizi* (*P*. *pachyrhizi*) and *Phytophthora sojae* (*P*. *sojae*). Expression intensities of PAMPs- and pathogen-treated samples were compared to untreated control experiments and log_2_-ratios were used for visualization. The following genotypes were used: LDX01-1-65 (weak PTI response), LD00-2817P (strong PTI response), Williams PI548631 (susceptible to *P*. *pachyrhizi*), PI459025B (resistant to *P*. *pachyrhizi*), Sloan (susceptible to *P*. *sojae*) and Conrad (resistant to *P*. *sojae*).
(XLSX)

S11 Table Expression pattern of legumes genes encoding CRKs with four DUF26 modules in different organs/tissues/treatments. *Glycine max* (Gm), *Medicago truncatula* (Medtr), and *Phaseolus vulgaris* (Phvu) expression data.
(XLSX)
