# Supplementary figures and images for: Genome-wide analysis of the soybean CRK-family and transcriptional regulation by biotic stress signals triggering plant immunity
Source: PLoS One. 2018 Nov 15;13(11):e0207438. doi: 10.1371/journal.pone.0207438 (PMC6237359; doi:10.1371/journal.pone.0207438)

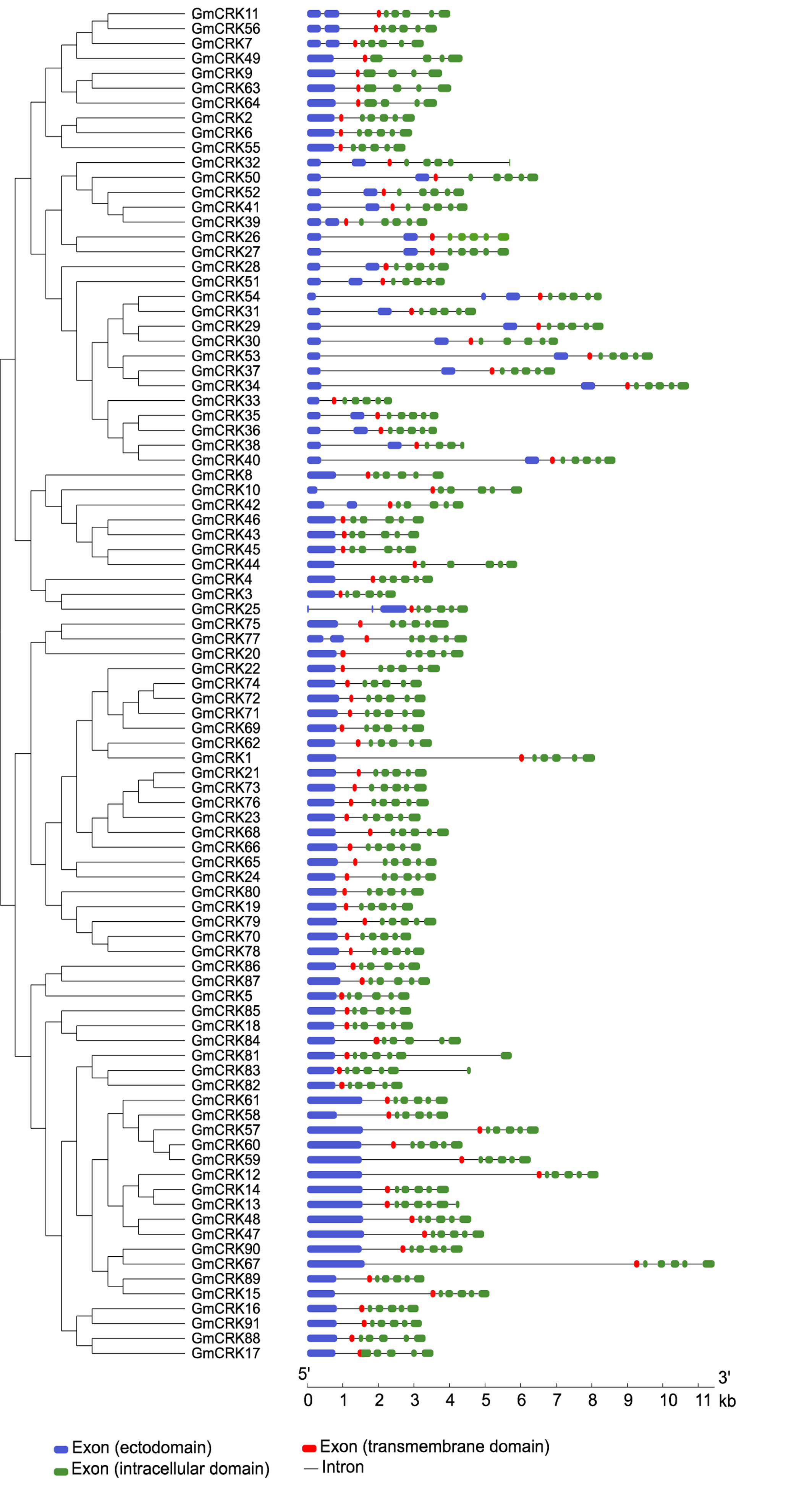

Supplement: S1 Fig — Lengths of introns and exons of GmCRKs genes are proportionally displayed based on the kilobase scale at the bottom of the figure. Yellow circles indicate GmCRKs with ectodomains containing 4 modules of DUF26. (TIF) [file pone.0207438.s001.tif]

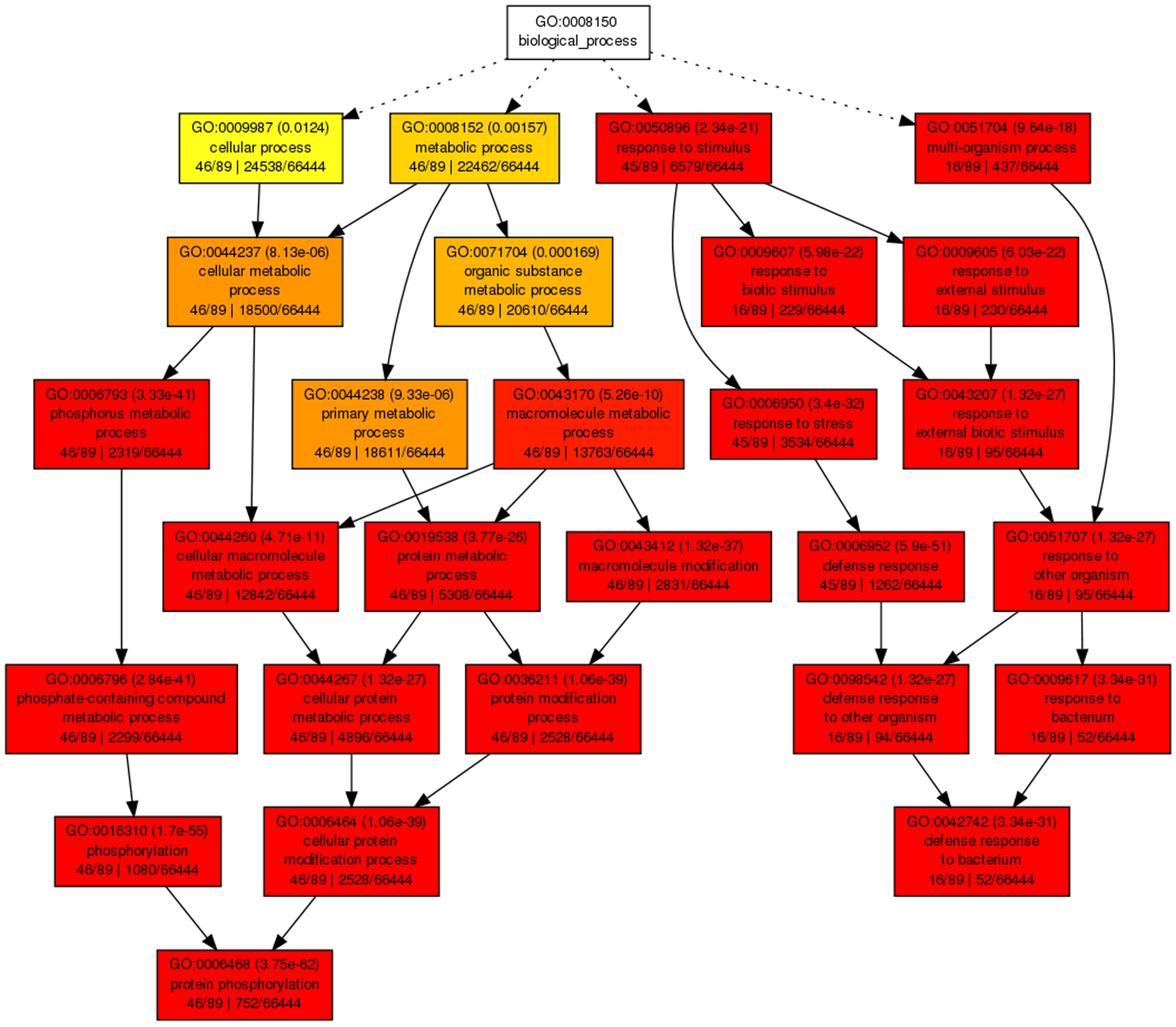

Supplement: S2 Fig — Statistically significant Biological Process GO terms were displayed with AgriGOv2.0. The information inside the boxes includes: GO term, adjusted p-value, GO description, item number mapping the GO in the query list and background, and total number of query list and background. (TIF) [file pone.0207438.s002.tif]

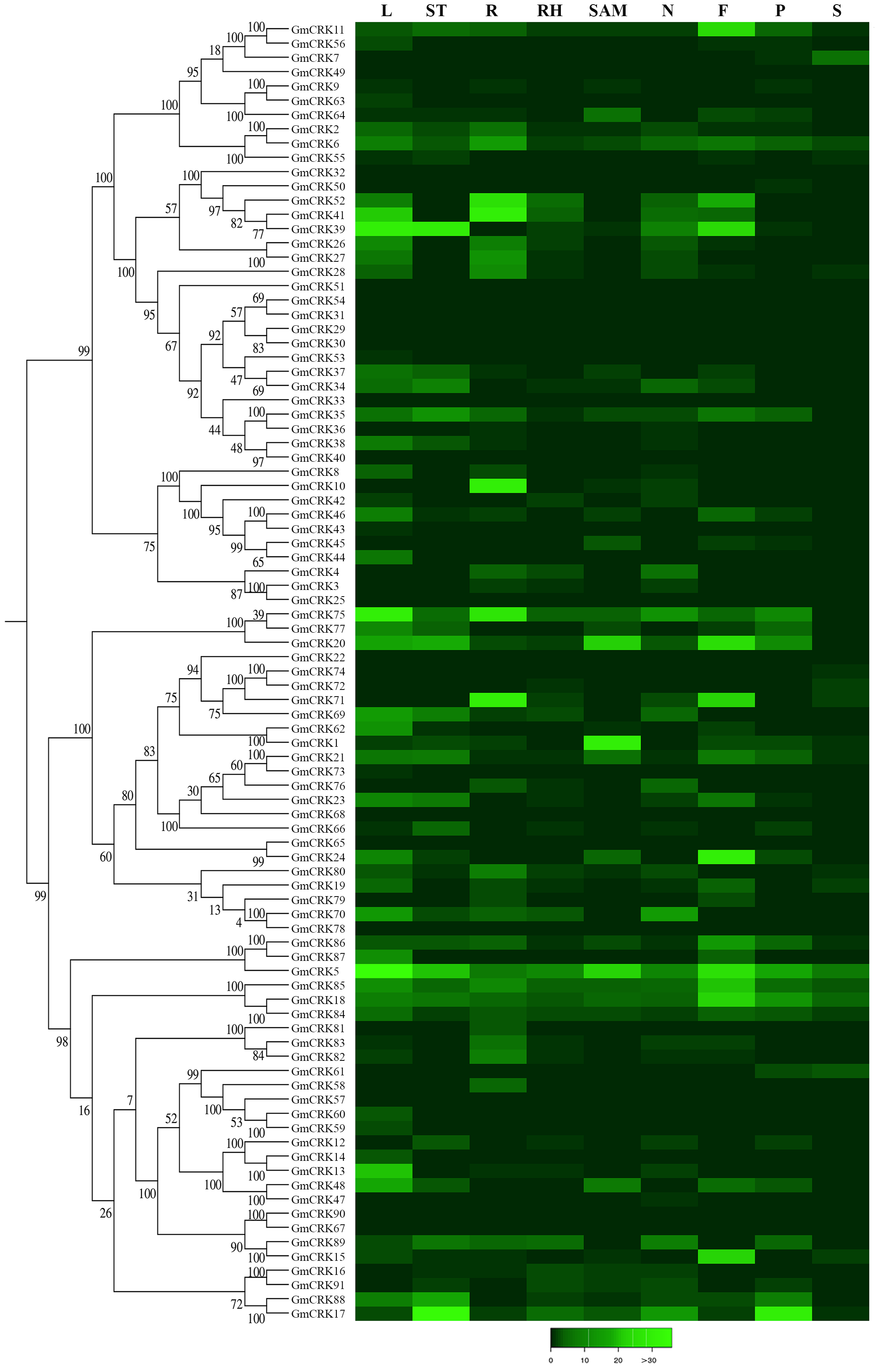

Supplement: S3 Fig — RNA-seq data was retrieved from Phytozome v12.1 and the fragments per kilobase of transcript per million mapped reads (FPKM) value of leaves (L), stem (ST), root (R), root hairs (RT), shoot apical meristem (SAM), nodules (N), flower (F), pod (P) and seed (S) were analyzed. The colored scale bar indicates gene expression level. (TIF) [file pone.0207438.s003.tif]

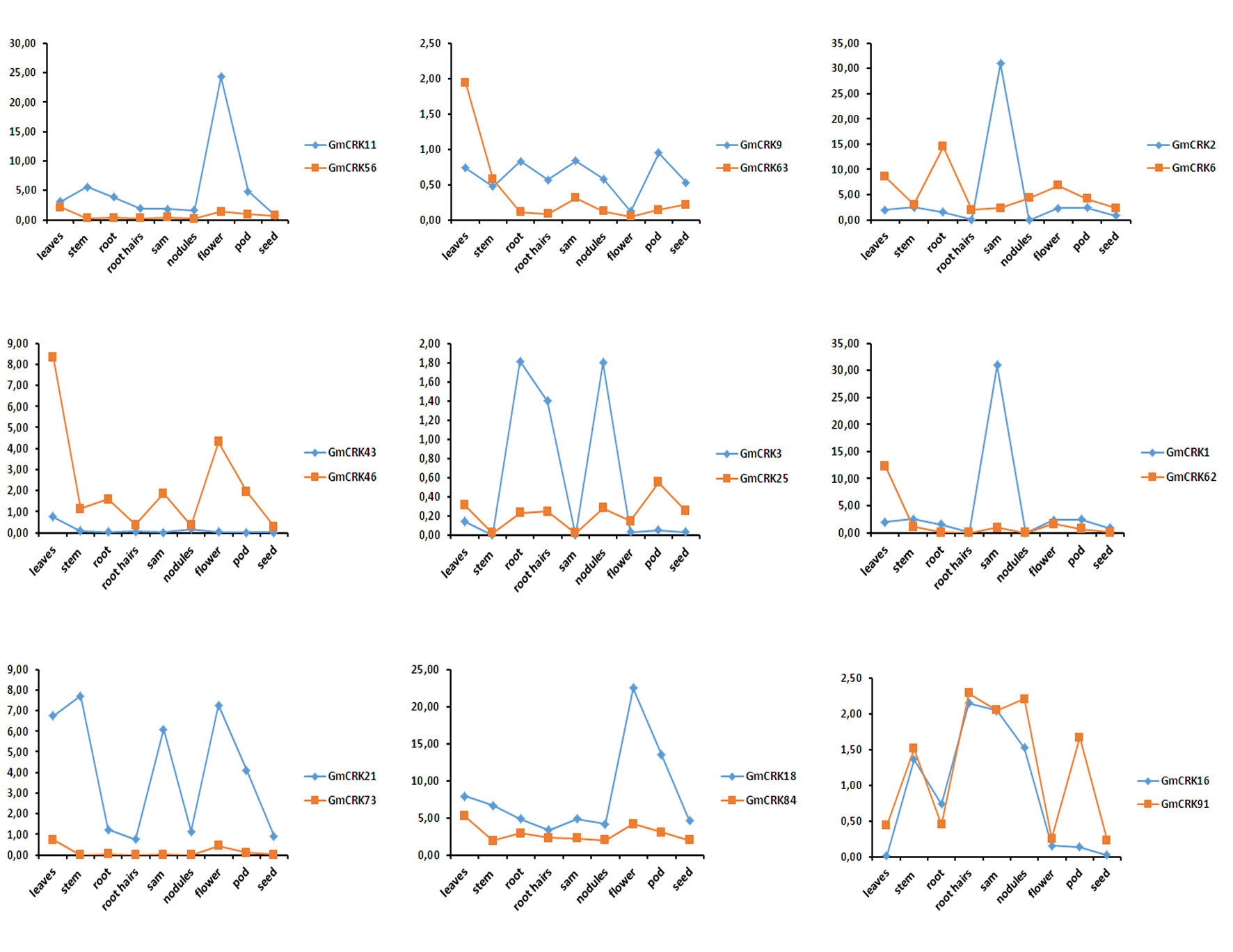

Supplement: S4 Fig — For each pair, the fragments per kilobase of transcript per million mapped reads (FPKM) values are presented as a line graph. (TIF) [file pone.0207438.s004.tif]

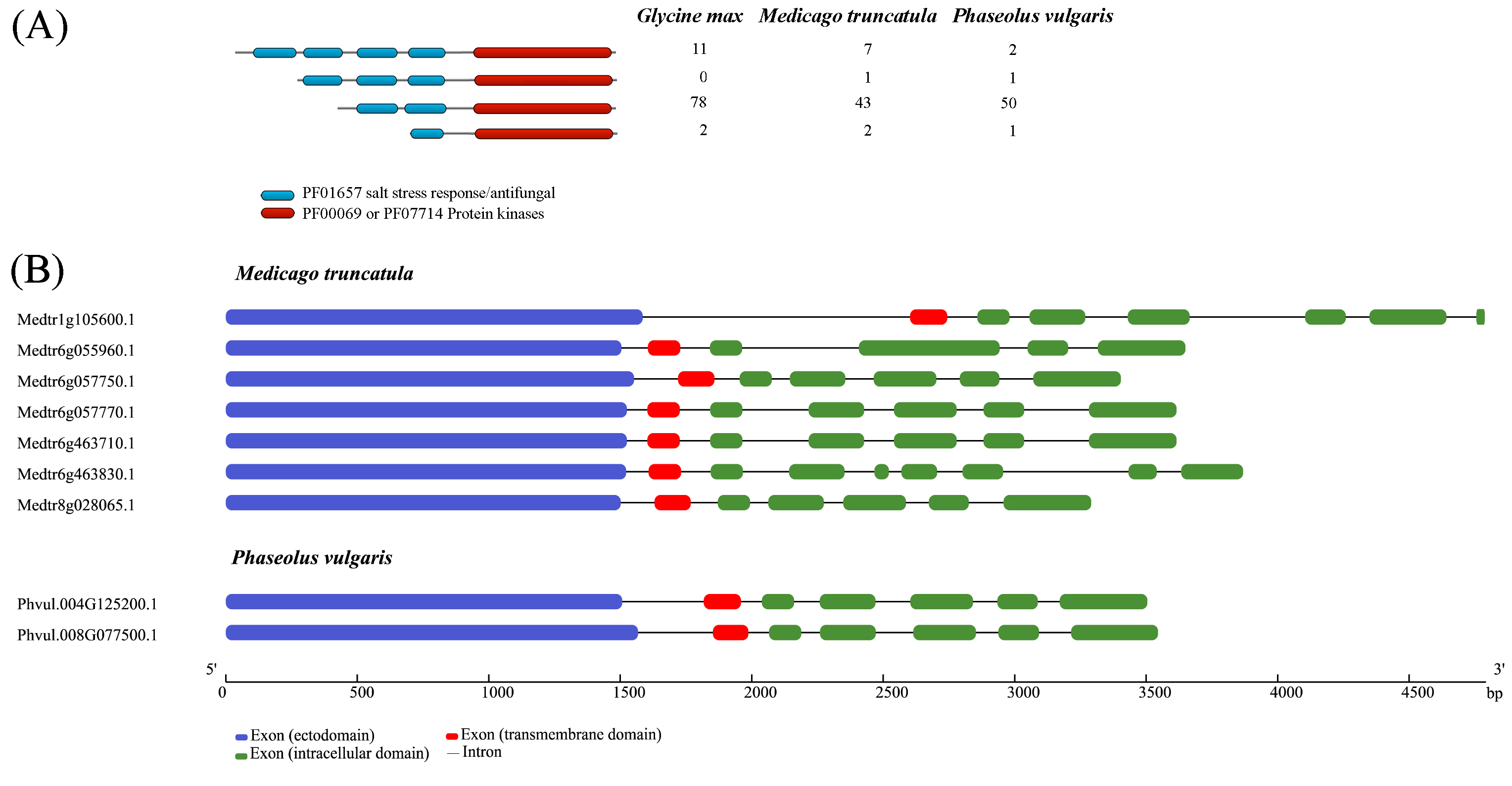

Supplement: S5 Fig — (A) Pfam domains present in legumes CRK predicted proteins. (B) Exon/intron structures of CRKs from Medicago truncatula and Phaseolus vulgaris, each one containing 4 modules of DUF26 within the ectodomains. (TIF) [file pone.0207438.s005.tif]

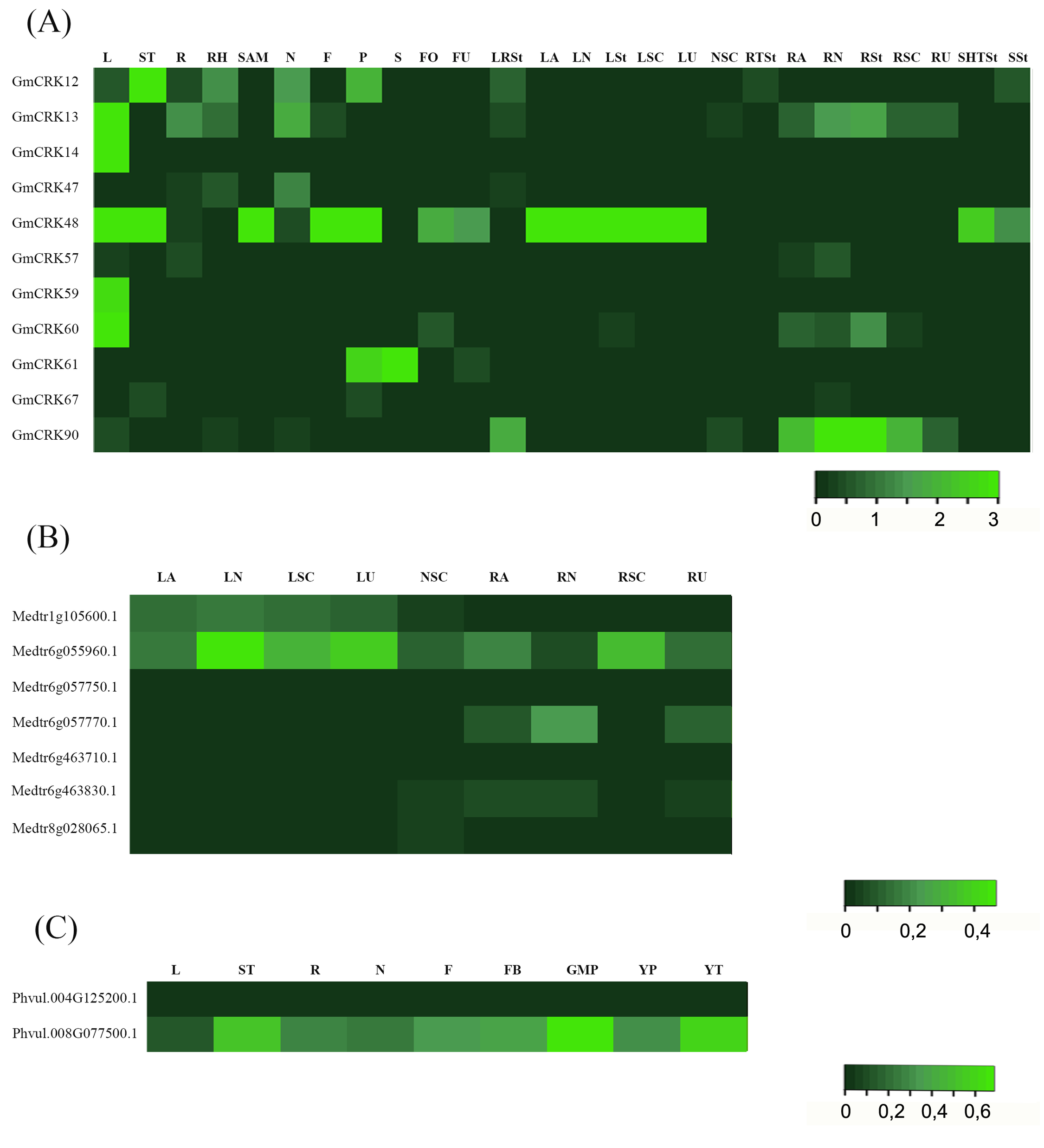

Supplement: S6 Fig — (A) Glycine max (Gm), (B) Medicago truncatula (Medtr), and (C) Phaseolus vulgaris (Phvu). RNA-seq data were collected from Phytozome v12.1 and fragments per kilobase of transcript per million mapped reads (FPKM) values of leaves (L), stem (ST), root (R), root hairs (RT), shoot apical meristem (SAM), nodules (N), flower (F), pod (P), seed (S), flower open (FO), flower unopen (FU), lateral root standard (LRSt), leaf ammonia (LA), leaf nitrate (LN), leaf standard (LSt), leaf symbiotic condition (LSC), leaf urea (LU), nodules symbiotic condition (NSC), root tip standard (RTSt), root ammonia (RA), root nitrate (RN), root standard (RSt), root symbiotic condition (RSC), root urea (RU), shoot tip standard (SHTSt), stem standard (SSt), flower buds (Fb), green mature pods (GMP), young pods (YP), young trifoliates (YT) are shown. Different colored scale bars for G. max, M. truncatula and P. vulgaris are shown, indicating gene expression level. (TIF) [file pone.0207438.s006.tif]
