# Supplementary material for: Genome-wide analysis of the soybean CRK-family and transcriptional regulation by biotic stress signals triggering plant immunity
Source: PLoS One. 2018 Nov 15;13(11):e0207438. doi: 10.1371/journal.pone.0207438 (PMC6237359; doi:10.1371/journal.pone.0207438)
Supplement: S4 Table — (PDF) [file pone.0207438.s010.pdf]

Supplementary Table 4. Primers used in RT-qPCR analysis.

Primer Name	Sequence (5´→3´)
GmCRK1 Forward	CCATAGTTGTGCCTACCGTTCTC
GmCRK1 Reverse	TCATCATCGTCTTCATCAGCGAG
GmCRK19 Forward	CCCTCTGAAGGAAAGAGCAACAC
GmCRK19 Reverse	CTCTTAGGAAAATGCAAACGGAG
GmCRK20 Forward	CCTATGATTATGCTGTCTGGTAGTGAG
GmCRK20 Reverse	ATTGGAGCCTTATTTGATGATCCTTG
GmCRK21 Forward	ATAGTAGAACTAGAAGCCTTTCAGAGC
GmCRK21 Reverse	AGGATATAGCTCAGTGATTGAAACCTC
GmCRK23 Forward	ATAGTTATTCTCTCACTCTCTCAGTGC
GmCRK23 Reverse	TCAGTTGATTTATTTGATCTTGTTTCCC
GmCRK24 Forward	TGCTACAGATGACTTCTCCGATTAC
GmCRK24 Reverse	ACTTCGTTCTCGAATTCTCTATCTCC
GmCRK28 Forward	CCCTCAAACTCTATTACTCAAACAGG
GmCRK28 Reverse	TTGATTGAGAAGTGGCAAAGTGC
GmCRK34 Forward	AGTAAGAGGGAAATGAGGAGGA
GmCRK34 Reverse	ATGAGAGAAAGTCACCAAACTCA
GmCRK42 Forward	CTTGCATGAACTCAACTTCTATTAGCC
GmCRK42 Reverse	GTGGTGTAGTTAAACCCTTTGCTTATG
GmCRK44 Forward	GGCTACATGGCTCCTGAGTACG
GmCRK44 Reverse	CTCTTTCCACAAATGATCTCCAGGAC
GmCRK55 Forward	TGAACAATAGAAGCAAAGCATCA
GmCRK55 Reverse	GGTTGTCATCCACCACTAGACA
GmCRK57 Forward	TCACCTTCAGGAAATAACTCACGA
GmCRK57 Reverse	TCTTTCTCGCCTTTCTTGTTATCAG

GmCRK61 Forward	CGTCACGAACAACTGTCTCT
GmCRK61 Reverse	GCTCTACATTAGTACTTTCAGCTCC
GmCRK69 Forward	AATGTAACTGCTTCAAGTTTTCAGACC
GmCRK69 Reverse	CTAAGAACTCTACCACCTATCTTGTCC
GmCRK73 Forward	CACCAACTATGACCAACAACACTTC
GmCRK73 Reverse	CACCAAAACAACACTAGCAACTGG
GmCRK75 Forward	GTGTACCATGAATGTAACAACCACTTC
GmCRK75 Reverse	GATTTCTTCGTGGGAATACACTTTGG
GmCRK77 Forward	ATACCGTTTTACTGATGCTTAACAGTG
GmCRK77 Reverse	ATAACCTGACCTTGTTACCTCTGAATG
GmCRK78 Forward	TGTTCAAGAAAACTTAGTCAACAGACC
GmCRK78 Reverse	CATTCATAGAATATGCAGGTTGAGAGG
GmCRK84 Forward	CACATGCCTGCTCGGATGAA
GmCRK84 Reverse	TCCAAGGGAGACGTTTGTGC
GmCRK87 Forward	AGAACTGAATCAAACATGCTGAAGTG
GmCRK87 Reverse	TCGAGGAATATCTCCACTAAGCGAC
EF1a Forward	GATTTCATGTAGCCGTAGCC
EF1a Reverse	ATTTAAGACATCCCTCCTCAG
