# Supplementary material for: Genome-wide analysis of the soybean CRK-family and transcriptional regulation by biotic stress signals triggering plant immunity
Source: PLoS One. 2018 Nov 15;13(11):e0207438. doi: 10.1371/journal.pone.0207438 (PMC6237359; doi:10.1371/journal.pone.0207438)
Supplement: S7 Table — (PDF) [file pone.0207438.s013.pdf]

Related to hormones

Cis Regulatory Element	Number of *GmCRK* genes	Description
CGTCA-motif/TGACG-motif	49	*cis*-acting regulatory element involved in the MeJA-responsiveness
TCA-element	43	*cis*-acting element involved in salicylic acid responsiveness
ABRE	39	*cis*-acting element involved in the abscisic acid responsiveness
GARE-motif/P-box	30	Gibberellin-responsive element
ERE	24	Ethylene-responsive element
TGA-element	10	Auxin-responsive element
AuxRR-core	5	*cis*-acting regulatory element involved in auxin responsiveness
CE3	1	*cis*-acting element involved in ABA and VP1 responsiveness
JERE	1	Jasmonate and elicitor-responsive element
TATC-box	1	*cis*-acting element involved in gibberellin-responsiveness

Related to biotic stress

Cis Regulatory Element	Number of *GmCRK* genes	Description
TC-rich repeats	51	*cis*-acting element involved in defense and stress responsiveness
W box/box S/GCC box	30	Wounding and pathogen responsiveness
WUN-motif	13	Wound-responsive element
MBSI/MBSII	11	MYB binding site involved in flavonoid biosynthetic genes regulation
EIRE/ELI-box3	8	Elicitor-responsive element
box E	2	*cis*-element for induction upon fungal elicitation

Related to abiotic stress

Cis Regulatory Element	Number of *GmCRK* genes	Description
HSE	50	*cis*-acting element involved in heat stress responsiveness
MBS	35	MYB binding site involved in drought-inducibility
LTR	11	*cis*-acting element involved in low-temperature responsiveness
DRE	1	*cis*-acting element involved in dehydration, low-temp, salt stresses
